# Emerging CRISPR Approaches for Countering Immune Evasion: Insight from Recent Studies

**DOI:** 10.3390/ijms27072930

**Published:** 2026-03-24

**Authors:** Sadam Abubakar, Latifat Abdulsalam, Lamin Fatty, Rimsha Kanwal, Muhammad Naeem, Irshad Ahmad

**Affiliations:** 1 Department of Bioengineering, King Fahd University of Petroleum and Minerals (KFUPM), Dhahran 31261, Saudi Arabia; g202315670@kfupm.edu.sa (S.A.); g202210360@kfupm.edu.sa (L.A.); 2 Department of Chemical Sciences, University of Padua, Via Marzolo 1, 35131 Padua, Italy; lamin.fatty@studenti.unipd.it; 3 Institute of Molecular Biology and Biotechnology (IMBB), The University of Lahore, Lahore 54000, Pakistan; rimshakanwal391@gmail.com; 4 Department of Cell and Developmental Biology, Weill Cornell Medicine, Cornell University, 1300 York Avenue, New York, NY 10065, USA; naeempunia@gmail.com; 5 Interdisciplinary Research Center for Membranes and Water Security, King Fahd University of Petroleum and Minerals (KFUPM), Dhahran 31261, Saudi Arabia

**Keywords:** cancer immunotherapy, immune evasion, gene editing, tumor infiltration, tumor microenvironment (TME)

## Abstract

Cancer immunotherapy has recently become an essential approach for treating cancer, showing considerable promise as a substitute for surgery, radiation therapy, and conventional chemotherapy. It primarily aims to boost the host’s natural defense system to combat cancer malignancies by utilizing components of immune checkpoint blockades (ICBs), mainly programmed cell death protein 1 (PD-1) and cytotoxic T-lymphocyte-associated antigen 4 (CTLA-4), along with elements of adoptive cellular therapies (ACTs) like Chimeric Antigen Receptor (CAR) therapy, T Cell Receptor (TCR) therapy and Tumor-Infiltrating Lymphocyte (TIL) therapy. However, cancer cells tend to undermine the effectiveness of cancer immunotherapeutic strategies by employing one or more immune evasion mechanisms. This review briefly highlights how key mechanisms of cancer immune evasion confer resistance to immunotherapy and how the Clustered Regularly Interspaced Short Palindromic Repeats/Cas9 (CRISPR)/Cas9 systems, as gene-editing tools, are poised to enhance cancer immunotherapy for treating challenging cancers. We emphasize that (CRISPR/Cas9) systems can be used to explore and positively alter the genes of the immune system, boosting the effectiveness of cancer immunotherapy by editing immune checkpoints, TILs, and CAR-T cells, and disrupting genes, facilitating tumors’ evasion of the immune system. Furthermore, we highlight the growing interest in emerging base editor technology to engineer natural killer (NK) cells to overcome NK-cell-based immunotherapy challenges, particularly human leukocyte antigens (HLA)-mediated limitations, and to engineer CAR-T cells for improved immunotherapy outcomes.

## 1. Introduction

### 1.1. Background on Cancer Immunotherapy

Cancer immunotherapy has gained significant attention as a safer and more effective alternative to conventional cancer treatment methods like surgery, radiation, and chemotherapy [1]. Traditional cancer therapies often fail to eliminate metastatic tumors, increasing the risks of recurrence. Additionally, they usually cause damage to surrounding tissues and systemic toxicities from chemotherapy [2,3].

Cancer immunotherapy seeks to stimulate or strengthen the host’s immune system to target and kill malignant tumors. Two principal strategies drive this approach: immune checkpoint blockade (ICB) and adoptive cellular therapies (ACTs). ICB employs immune checkpoint inhibitors (ICIs) to restore T cell activity, primarily by targeting cytotoxic T-lymphocyte-associated antigen 4 (CTLA-4)/B7-1/B7-2 and programmed cell death protein 1/ programmed death ligand 1 (PD-1)/(PD-L1) on T cells and cancer cells, key regulators of immune response [4,5]. Conversely, ACTs depend on T cells, either autologous or genetically engineered, to enhance their tumor-targeting capabilities. Major ACT strategies include chimeric antigen receptor (CAR) therapy, T-cell receptor (TCR) therapy, and tumor-infiltrating lymphocytes (TIL) therapy. Although these strategies show promising results in treating specific cancer types, many patients remain inadequately treated due to both intrinsic and acquired tumor resistance. Immunotherapeutic approaches face several limitations, including off-target effects, toxicities, immunosuppressive barriers, and challenges in standardizing manufacturing [6].

A significant obstacle to effective immunotherapy is the ability of tumors to evade the immune system, which diminishes the effectiveness of the treatment [7]. Immune evasion involves a range of strategies through which tumors escape targeting and subsequent destruction by the immune system, even when reactivated by immunotherapy [8]. One common approach involves minimizing the expression of antigens on cancer cells [8]. Tumor cells may also suppress the immune system’s response to tumors by enhancing the expression of immune checkpoint proteins, like PD-L1, within the tumor microenvironment (TME) [9]. Moreover, tumor cells may further hinder immune cell function by creating a microenvironment rich in specific metabolites and signaling factors, or by depriving immune cells of essential nutrients [8].

Consequently, a thorough understanding of tumor immune evasion mechanisms and the development of strategies capable of precisely modulating immune and tumor-associated genes are necessary to improve therapeutic efficacy.

### 1.2. Gene Editing in Modern Medicine

Genetic engineering emerged in the 1970s, resulting in significant advancements in gene-editing technologies. This development enabled unprecedented insights into how single-gene products contribute to disease mechanisms. Since then, gene editing has been extensively utilized in research, medical science, and biotechnology [10,11]. The process typically involves producing double-stranded break (DSB) in DNA using engineered or bacterial nucleases. These breaks can be repaired via homology-directed repair (HDR), which promotes precise integration when a template is available, or through non-homologous end joining (NHEJ), an imprecise process that often causes gene disruptions [12,13].

In gene replacement, addition, or inactivation, homologous recombination (HR) is preferred since it utilizes homologous DNA as a template for repair. However, its low efficiency in mammalian cells and many model organisms limits its application [14]. Since DSBs can stimulate HDR at specific genomic loci, a targeted nuclease strategy was developed to introduce site-specific DSBs, thereby enhancing the specificity of genome editing [11]. This method introduces exogenous DNA aligned with the break site, thereby enabling site-specific repair [11,15].

Conversely, the NHEJ mechanism is often deemed error-prone, typically resulting in deletions (indels) or insertions at DSB sites, which can potentially cause frameshift mutations and lead to truncated proteins [16,17]. NHEJ is easier to implement than HR, as it does not require a template and relies less on the efficiency of the repair process [18]. As a result, NHEJ is widely used to inactivate one or more genes in cell lines [19].

Genome editing has utilized tools, including Zinc Finger Nucleases (ZFNs) and Transcription Activator-Like Effector Nucleases (TALENs). However, the clustered regularly interspaced short palindromic repeats (CRISPR) and associated Cas9 nuclease system has outperformed them in several aspects. Some notable superior properties of the CRISPR/Cas9 system compared to other gene-editing tools include operability, scalability, and flexibility [20,21,22]. As such, the CRISPR/Cas9 system is arguably the most advanced gene-editing tool in the history of genetic modification. The CRISPR/Cas system originates from the bacterial adaptive immune system. It was brought to the limelight in 2013 when it was initially utilized as an instrument for mammalian genome editing [23,24]. The CRISPR/Cas9 technology is a robust, programmable instrument for editing eukaryotic genomes. Its programmable nature enables precise editing of eukaryotic genomes, including pre-transcriptional genomic sequence modification and transcriptional and epigenetic alterations [11,25].

While bacteria evolved CRISPR as an adaptive immune defense system over evolutionary timescales, researchers now use it to modulate the immune systems of mice against cancer cells. They are exploring genes linked to tumor immune evasion as potential targets for cancer immunotherapy and disrupting specific genes to enhance the effectiveness of this therapy. These developments have positioned CRISPR/Cas9 systems as a powerful tool for improving cancer immunotherapeutic strategies.

### 1.3. CRISPR/Cas9 in Cancer Immunotherapy and the Scope of This Review

Emerging gene-editing techniques, particularly the CRISPR/Cas9 system, are well-positioned to address the challenges of cancer immune evasion, which hamper the success of cancer immunotherapy.

The CRISPR/Cas system is already recognized as a versatile tool for combating immune evasion, primarily due to its diverse applications. In cancer research, the system has been extensively utilized to study the mechanisms of tumor progression, identify regulators of immune escape, and engineer immune cells with enhanced therapeutic potential [26,27,28]. CRISPR/Cas9 has also gained traction for its remarkable capacity to manipulate and regulate several gene functions by simultaneously targeting multiple loci [23].

Consequently, CRISPR/Cas9-based genetic engineering is considered among the most promising approaches to treating cancer, viral infections, cardiovascular problems, and immunological and genetic diseases [29]. Furthermore, the efficient genomic manipulation ability of CRISPR/Cas9 has enabled the creation of various animal cancer models, a more profound investigation of epigenetic regulation, and, significantly, the genetic manipulation of immune and cancer cells in cancer immunotherapy [1]. Importantly, CRISPR-based engineering has emerged as a critical tool for enhancing cell-based immunotherapies, including the modification of TILs, CAR-T cells, and natural killer (NK) cells.

While several previous reviews have focused on the application of CRISPR technologies in immune cell engineering, particularly in the context of CAR-T cell development or checkpoint-targeting, the present review aims to provide a broader, more integrated perspective. Specifically, we emphasize the interconnected roles of tumor-intrinsic and immune-cell-intrinsic regulators in shaping anti-tumor immunity. Effective immunotherapy requires not only the functional optimization of immune effector cells but also the disruption of tumor-intrinsic mechanisms that enable immune evasion [30]. CRISPR-based functional genomics has been instrumental in uncovering these regulatory networks, revealing key tumor pathways that influence immune recognition and response.

Across multiple CRISPR screening studies, several shared mechanistic themes have emerged. Notably, genes involved in interferon-γ (IFNγ) signaling, antigen processing and presentation, and immune-cell fitness and persistence repeatedly appear as critical determinants of therapeutic response. Disruption of IFNγ pathway components can render tumor cells resistant to immune-mediated killing, while alterations in antigen presentation pathways, including major histocompatibility complex (MHC) regulation, can impair T cell recognition [31,32,33,34]. Conversely, engineering immune cells to enhance metabolic resilience, reduce exhaustion, or remove inhibitory checkpoint signaling has been shown to improve their cytotoxic activity and persistence within the TME.

In this review, we discuss how CRISPR-based technologies are used to both identify and manipulate genes that regulate tumor immune evasion and immune-cell function. We first examine key immune evasion mechanisms, CRISPR-mediated screening of immunomodulatory genes, and immune manipulation strategies. We then explore strategies for engineering immune effector cells, including CAR-T cells, TILs, and NK cells, to enhance their therapeutic efficacy. In addition, we highlight emerging genome-editing approaches, including base-editing technologies, that offer improved precision and safety for immune-cell engineering. Finally, we discuss key translational challenges associated with CRISPR-based immunotherapy and outline future directions for integrating genome engineering into next-generation cancer treatment strategies.

## 2. Immune Evasion Mechanisms in Cancer

Our review focuses on CRISPR-based strategies to address immune evasion. Accordingly, this section provides a concise, selective discussion of immune evasion mechanisms directly relevant to CRISPR interrogation; comprehensive details of these mechanisms have recently been provided by Li et al. (2024) [35]. We therefore focus on mechanisms that are not only genetically modifiable but also amenable to experimental interrogation, including tumor heterogeneity, antigen instability, immunosuppressive microenvironments, and checkpoint signaling. Together, these examples highlight why conventional approaches often fail and why CRISPR-based functional genomic approaches are essential to dissect and therapeutically target adaptive immune resistance for enhanced immunotherapeutic outcomes.

### 2.1. Tumor-Intrinsic Variability and Antigen Instability

Tumor intrinsic variability represents a significant obstacle to the effectiveness of cancer immunotherapy. Cancer cells within a given tumor may differ in antigen expression, genetic composition, and immune susceptibility. These key traits largely influence the resistance of the cancer cell subpopulations under therapeutic pressure. Tumor heterogeneity has therefore been recognized as a key driver influencing tumor evolution, progression, and metastasis. As such, a deeper understanding of clonal complexities across tumors is necessary to elucidate how this phenomenon contributes to tumor progression and immune evasion, and to inform more effective treatment strategies [36].

In ACTs such as CAR-T cell treatments, tumor heterogeneity can drive the emergence of antigen-negative or antigen-low variant subclones. For instance, a single-cell profiling study identified pre-existing CD19-negative subclones within a heterogeneous tumor population in a patient with B cell acute lymphoblastic leukemia (B-ALL) [37]. Thus, these rare pre-existing CD19-negative leukemic cells were selected during treatment and caused disease relapse after CAR-T cell therapy.

A similar pattern of antigen downregulation or loss has been observed in other B cell malignancies. For example, in multiple myeloma (MM), reduced expression or loss of B cell maturation antigen (BCMA) has been observed following BCMA-targeted CAR-T therapies, thereby limiting treatment efficacy and promoting disease relapse. These observations have motivated the development of strategies to mitigate antigen escape in CAR-T therapies. One such strategy involves dual targeting of BCMA and another antigen (CD38) expressed by multiple myeloma (MM) cells. The bispecific CAR (BM38 CAR) has been shown to improve the efficacy of CAR-T therapy in patients with relapsed or refractory multiple myeloma (RRMM) [38].

At the molecular level, antigen instability in cancer cells may also arise through genetically driven processes, particularly point mutations and alternative splicing [39]. For instance, alternative splicing of CD19 can generate isoforms lacking critical epitopes or transmembrane domains required for CAR recognition. This phenomenon renders tumor cells invisible to CAR-T cells despite ongoing gene transcription, causing immune evasion and resistance to CAR-T therapy [40]. In addition to antigen loss, cancer cells within a tumor can evade immune recognition by masking the target antigen. This phenomenon was observed in leukemia, in which leukemic cells aberrantly expressed the CAR construct that binds the targeted antigen. Therefore, masking of the target antigen allows tumor cells to evade recognition by therapeutic CAR-T cells, thereby promoting resistance to CAR-T therapy [41,42].

Collectively, tumor heterogeneity, antigen loss, and antigen masking demonstrate that complex and often unpredictable tumor-intrinsic mechanisms govern immune evasion. These features limit the effectiveness of single-target approaches and necessitate robust strategies for the systematic identification of genetic drivers of antigen escape and adaptive resistance. CRISPR is emerging as an indispensable engineering tool with the potential to address the issues of antigen instability, antigen masking, and tumor heterogeneity. We have highlighted how CRISPR-based approaches are improving the robustness of CAR-T cells for targeting cancer cell antigens and destroying cancer cells. There are also emerging CRISPR approaches for multiplex targeting of resistance determinants and antigens to address immune resistance through antigen downregulation or variability.

### 2.2. The Immunosuppressive Tumor Microenvironment and Cellular Interactions

Tumor-intrinsic mechanisms do not solely govern immune evasion. Immune evasion can also be driven by coordinated interactions between malignant cells and the TME. The TME has increasingly emerged as a significant barrier to the success of immunotherapy, primarily through immune evasion and immune resistance. Significantly, the dynamic properties of the TME are shaped not only by solid tumor cells but also by nonmalignant cellular and structural components [43].

Tumor immune inhibition or evasion is often not driven by a single suppressive mechanism. Instead, it arises from the coordinated functions of various immune and stromal components within the TME. Regulatory lymphocytes, suppressive myeloid populations, and stromal elements can collectively dampen immune cell activation, restrict tumor infiltration, and impair effective antitumor responses [43,44]. A significant challenge in targeting these processes lies in their functional interconnections. This makes an inhibition of one pathway being compensated for by others, thereby contributing to the persistence of immune resistance within the TME.

The interdependence of these suppressive networks suggests that effective strategies to overcome TME-mediated immunotherapy resistance require novel immunotherapeutic approaches. Accordingly, CRISPR-based approaches enabled thorough interrogation of TME components and coordinated targeting of multiple regulatory pathways, rather than inhibiting isolated pathways, thereby providing a mechanistic framework for overcoming TME-mediated immune resistance and enhancing the outcome of cancer immunotherapy.

### 2.3. Immune Checkpoint Signaling and Inhibitory Networks

Immune checkpoint signaling has been established as a central mechanism of tumor resistance to antitumor immune responses. Although the use of ICIs represents a turning point in cancer therapy, patients treated with ICIs often derive limited or no clinical benefit. Even among patients who initially experience a favorable outcome with ICI treatment, disease relapse due to acquired resistance is common [45].

Growing evidence indicates that a single molecular alteration does not drive resistance to ICIs; rather, it arises from dynamic, complex, and interconnected regulatory processes. Resistance mechanisms to ICIs are broadly classified as intrinsic or extrinsic. Intrinsic resistance primarily results from alterations in gene expression, cell signaling pathways, immune recognition, and immune cell effector functions that ultimately lead to resistance. In contrast, extrinsic resistance, which occurs outside tumor cells (the tumor microenvironment), is mediated by both immunological and non-immunological interactions that collectively restrict immune activity [45].

Importantly, intrinsic and extrinsic resistance mechanisms do not operate independently. As such, they are viewed as operating simultaneously and compensating for one another. This functional interdependence explains the limitations of receptor-focused inhibitory pathways and the mechanisms that enable the tumor to adapt to alternative inhibitors and regulatory networks [45].

The combined influence of tumor-intrinsic variability, TME-dependent immune suppression, and immune checkpoint-mediated inhibition demonstrates that immune evasion is not governed by a single pathway or static phenomenon. Immune evasion is therefore governed by dynamic, interconnected networks operating across tumors and immune cells.

As such, immune resistance cannot be fully understood through descriptive or correlational analyses alone. Instead, it requires a tool for systematic functional interrogation to distinguish causal drivers of resistance from secondary associations. This positions CRISPR-based technologies for high-resolution mapping of immune evasion determinants, enabling not only genome-scale discovery but also precise mechanistic validation. CRISPR-enabled functional insights provide the foundation for rationally designed, multiplex immunotherapeutic approaches capable of overcoming not only checkpoint-mediated cancer immune resistance but also broader immune evasion networks [46].

## 3. The CRISPR/Cas9 Structure and Mechanisms

### 3.1. Overview of the CRISPR/Cas9 Mechanisms: Guide RNA, Cas9 Enzyme, and DNA Targeting

The sequences known as clustered regularly interspaced short palindromic repeats (CRISPRs) were first identified in 1987 in *Escherichia coli* and subsequently discovered in various other bacterial species [47]. Their function was later discovered to be part of the adaptive immune mechanisms of archaea and bacteria, in which they defend against invading genetic elements, such as bacteriophages, through RNA-guided DNA cleavage [48,49,50]. The CRISPR Cas system operates through three sequential stages: acquisition, transcription, and interference [50,51]. Owing to its simplicity, precision, and adaptability, the CRISPR/Cas9 has been thoroughly studied for its structure, functionality, and application as a gene-editing tool in both experimental and clinical settings since its discovery [52].

Interestingly, scientists are more attracted to the type II CRISPR/Cas9 system, particularly for human genome engineering clinical applications [53]. CRISPR/Cas9 relies on the *Streptococcus pyogenes* single Cas protein (SpCas9), which has the key distinguishing feature of targeting specific DNA sequences, contributing to its widespread adoption [53]. However, while SpCas9 remains the most utilized variant, other Cas proteins, such as Cas12, Cas13, and Cas14, have emerged with unique properties, including altered PAM requirements, RNA-targeting capabilities, and smaller sizes, thereby broadening the genome editing and engineering platforms [54].

In the type II system, a specific DNA segment from the invading genetic element is integrated into the host (archaeal/bacterial) CRISPR locus. This integrated DNA segment is termed a spacer [55]. The CRISPR locus is transcribed into a long precursor CRISPR RNA (pre-crRNA), which is subsequently processed into short CRISPR-derived RNAs (crRNAs). These crRNAs pair with a trans-activating CRISPR RNA (tracrRNA) to form a dual-complex RNA structure that guides the Cas9 protein to a specific target DNA. For genome-editing applications, the crRNA and tracrRNA are commonly fused into a single-guide RNA (sgRNA) [48].

Within the sgRNA, a spacer region is complementary to the target DNA sequence, while the scaffold region forms a hairpin structure recognized by Cas9. Once loaded onto Cas9, the sgRNA directs the protein to the complementary sequence on the target DNA, provided that an appropriate protospacer adjacent motif (PAM) is present immediately adjacent to the target site [56,57,58]. Upon target recognition, the Cas9 protein induces a site-specific double-strand break (DSB) through the coordinated action of its RuvC-like and HNH nuclease domains [59,60,61]. Thus, the CRISPR/Cas9 system fundamentally consists of two core elements: a single-guide RNA and a Cas9 endonuclease [52].

**Figure 1:**
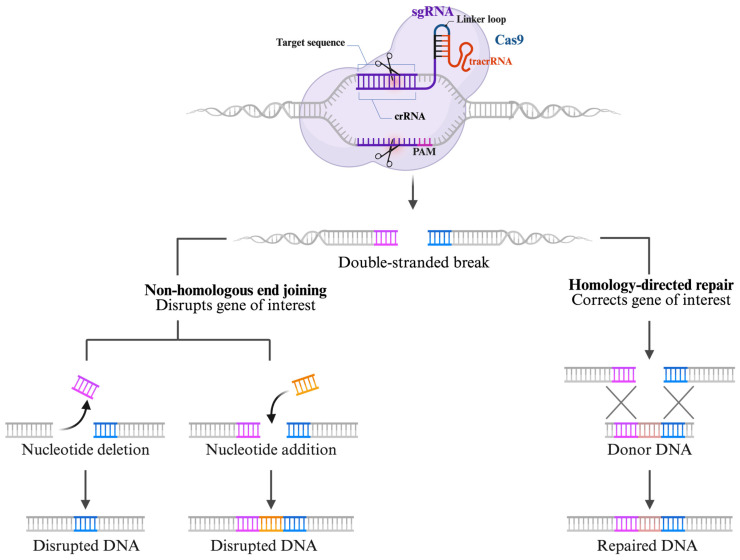
CRISPR/Cas9-driven DSBs and the two DSB repair mechanisms. The guide RNA (sgRNA) first pairs with the target DNA sequence, resulting in a site-specific DSB created by the Cas9 endonucleases. The repair of the DSBs occurs through either NHEJ or HDR mechanisms. Created in Biorender. S. Abubakar et al. (2026) www.BioRender.com (accessed on 3 March 2025).

Following DSB formation, DNA repair is mediated primarily through non-homologous end joining (NHEJ) or homology-directed repair (HDR) (Figure 1). NHEJ is an error-prone repair mechanism that frequently introduces insertions or deletions, whereas HDR enables precise DNA sequence correction or insertion using a homologous donor template [61,62]. However, HDR is restricted to specific phases of the cell cycle, predominantly the S and G2/M phases, which has motivated efforts to enhance its efficiency through cell-cycle modulation strategies [63].

Beyond genome cleavage, the CRISPR/Cas9 platform has been adapted for transcriptional regulation by using catalytically inactive Cas9 (dCas9), enabling targeted gene activation (CRISPRa) or repression (CRISPRi) without inducing DNA DSBs [64]. Several RNA-guided CRISPR systems have progressed from fundamental research into various stages of preclinical and clinical development, underscoring their broad applicability in genome engineering and therapeutic intervention [52].

The CRISPR/Cas9 system, owing to its programmable and modular architecture, is now a platform that enables precise gene disruption, correction, activation, or repression within complex cellular systems. This unique versatility makes CRISPR systems well-suited to dissecting the genetic determinants of tumor immune evasion and to engineering immune cells for enhanced antitumor function, as discussed in the subsequent sections.

### 3.2. The CRISPR Functional Screens of Immunomodulatory Genes

**Figure 2:**
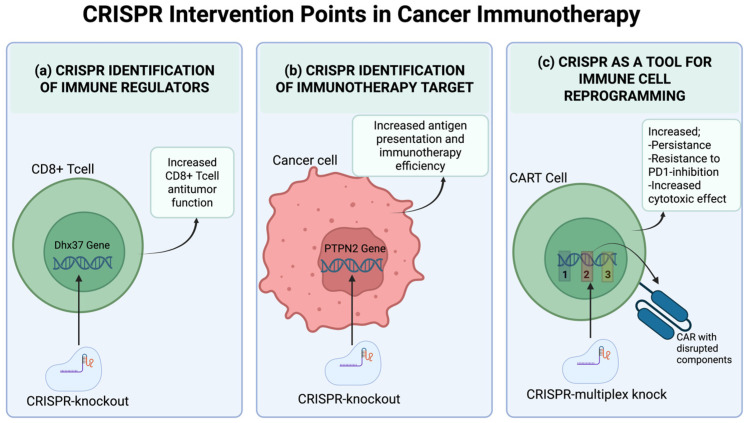
CRISPR intervention points in cancer immunotherapy. This figure illustrates the intervention points and purposes at which CRISPR technology can be applied in cancer immunotherapy. (**a**) CRISPR knockout of the *Dhx37* gene enables the discovery of its regulatory function because its ablation increased CD8+ T cell antitumor function. (**b**) CRISPR knockout could lead to the discovery of novel immunotherapy targets. Disruption of the *PTPN2* gene increased antigen presentation, potentially reversing cancer cells’ downregulation of antigen presentation, thereby enhancing immune cell targeting and cancer immunotherapy. (**c**) CRISPR multiplex knockout reprogrammed CAR-T cells to increase persistence, cytotoxicity, and resistance to PD-1 inhibition. Created in Biorender. S. Abubakar et al. (2026) www.BioRender.com (accessed on 22 January 2026).

A logical strategy to improve the efficacy of cancer immunotherapy involves mapping genes that modulate anti-tumor immunity. CRISPR/Cas9 systems can be used to induce loss-of-function (LOF) or gain-of-function (GOF) perturbations in both tumor and immune cells, thereby identifying relevant immunomodulatory genes that shape therapeutic response Collectively, functional CRISPR screening approaches have enabled the systematic identification of both tumor-intrinsic and immune cell-intrinsic regulators of antitumor immunity that shape therapeutic responses (Figure 2).

For clarity, this subsection is organized according to the cellular compartment interrogated (tumor-intrinsic and immune-cell-intrinsic regulators), as well as based on the screening modality employed.

#### 3.2.1. Tumor Intrinsic CRISPR Screens

Genome-wide LOF screening in tumor cells has enabled the identification of genes that directly influence immune recognition and cancer immune evasion and resistance.

For example, one in vivo CRISPR/Cas9 screen was used to identify new immunotherapy targets while also supporting the roles of established immune evasion molecules. In this study, pooled LOF screening in melanoma cells further supported the roles of PD-L1 and CD47 and demonstrated that disruption of interferon-γ signaling diminishes the effectiveness of immunotherapy. Additionally, the protein tyrosine phosphatase non-receptor type 2 (*PTPN2*) was identified as a new target for cancer immunotherapy. The deletion of *PTPN2* enhanced interferon-γ-dependent growth suppression and increased antigen presentation [65].

Similarly, a recent study proposed that tyrosylprotein sulfotransferase-2 (*TPST2)* be considered a novel target in immune checkpoint therapy, based on its role revealed by genome-wide CRISPR screening. The *TPST2* mediates the sulfation of IFNγ receptor 1 at Y397, thereby disrupting IFNγ-dependent signaling. Conversely, depletion of *TPST2* expression stimulates IFNγ-dependent signaling and improves antigen presentation. The inverse relationship between *TPST2* expression and antigen presentation, as observed in RNA sequencing data, further supports its role as a suppressor of anti-tumor immunity [31].

More recent studies have employed fluorescence-activated cell sorting (FACS)-based genome-wide screens in multiple murine cancer cell lines. Using a LOF approach, *DNAJC13* was identified as a regulator of CD47 expression in the B16 (melanoma), EMT6 (breast carcinoma), and MC38 (colon adenocarcinoma) cell lines. Functional validation demonstrated that *DNAJC13* ablation reduced CD47 surface expression. Consistent with these findings, *DNAJC13*-deficient cells exhibited increased susceptibility to macrophage-mediated phagocytosis, underscoring the role of *DNAJC13* in immune evasion. Moreover, a significant reduction in tumor burden was observed when *DNAJC13*-knockout tumors were treated with CD47 blockade [66].

These tumor-intrinsic LOF screens demonstrate how CRISPR allows for the systematic identification of genes that influences antigen presentation, checkpoint regulation, and immune susceptibility within cancer cells.

#### 3.2.2. Immune-Cell-Intrinsic CRISPR Screens

Beyond tumor-intrinsic regulators of immune sensitivity, CRISPR screening has also enabled the discovery of immune-cell-intrinsic factors that dampen immune-cell effector functions and ultimately reduce the efficacy of cancer immunotherapy.

Most immune-cell-intrinsic CRISPR screens have been conducted using the LOF CRISPR approaches.

For example, another study employs CRISPR-based genome-scale screening in CD8^+^ T cells to identify genes that influence immunotherapy outcomes. The screening study, in addition to confirming the relevance of PD-1 and Tim-3 as established immunotherapeutic targets, also highlights additional or novel genes with immunotherapeutic roles that had not been previously linked to T cell-mediated anti-tumor responses. Using similar LOF experiments, a *Dhx37* knockout has been shown to increase the efficacy of CD8^+^ T cells against a specific breast cancer cell type in an in vivo study. It has also been demonstrated that *Dhx37* plays a key role in the arrest of several immunotherapeutic functions of T cells against cancer cells, thereby confirming *Dhx37* as a novel regulator of CD8^+^ T cells. Taken together, these studies demonstrate that CRISPR functional screening can, in addition to supporting the role of well-known immune checkpoint molecules, reveal novel and less obvious regulators that limit the effectiveness of anti-tumor T cell responses [67].

Another study conducted genome-wide CRISPR/Cas9 knockout screens to identify genes that suppress the overall fitness of CD8^+^ T cells across three key stimulation modes. This study identified several regulators that suppress T cell fitness and antitumor function. It has been demonstrated that *Dap5* negatively regulates T cells, as its ablation not only increases T cell translation but also improves T cell fitness under various stimulations. Additionally, disruption of *Icam1*-dependent T cell clustering amplifies T cell effector functions following both intense and acute stimulation. Unlike *Icam1*, which is influenced by both chronic and acute stimulation, *Ctbp1* disruption promoted T cell persistence only under chronic stimulation. Taken together, these findings reveal how CRISPR/Cas9-based screens uncover T cell fitness regulators that can act individually or in combination to modulate T cell antitumor properties [68].

In addition to LOF screening for novel immunotherapeutic targets, GOF approaches such as CRISPR activation (CRISPRa) are also used. For example, a group of scientists engineered a CRISPRa platform that specifically targeted the knock-in or overexpression of a particular gene in CD8^+^ T cells for CAR-T engineering. In this study, overexpression or knock-in of *PRODH2* not only enhanced CAR-T-based killing by engineered CAR-T cells but also improved efficacy across multiple cancer models tested. Engineering *PRODH2* in CAR-T cells revealed its influences on multiple CAR-T cell functions, particularly mitochondrial, metabolic, and immunological functions, in combating cancer cells. The results of this study show that CRISPRa, which facilitates GOF, can be a powerful tool for uncovering genes that could be engineered to improve the effectiveness of cancer immunotherapy [69].

Together, these studies demonstrate that CRISPR-based functional screening extends beyond validating well-established immune checkpoints to the systematic discovery of previously unrecognized regulators of immune sensitivity, resistance, and effector fitness. Importantly, these regulators operate across both tumor and immune compartments, reinforcing the view that immune evasion is not only genetically encoded but a context-dependent process. The CRISPR enabled genome-scale perturbations with functional readouts, which has transformed immunotherapy development from hypothesis-driven target selection to a more robust unbiased identification of actionable regulatory networks.

#### 3.2.3. The CRISPR-Mediated Reprogramming of Immune Cell Function to Overcome Immune Suppression

In addition to discovery-driven screening, CRISPR/Cas9 technology is emerging as a powerful tool for the functional reprogramming of immune cells. Through intentional genetic perturbations, reprogrammed immune cells can be endowed with enhanced capabilities to overcome barriers imposed by the tumor microenvironment. Hence, CRISPR/Cas9 technology extends beyond functioning as a genetic engineering tool by enabling systematic CRISPR-mediated perturbations, interrogation, and modification of the immune-intrinsic regulatory pathway that dampens antitumor activity.

Recent functional CRISPR studies in primary T cells have further highlighted that immune cell dysfunction is driven not only by intercellular interactions but also by genetically encoded networks that regulate T cell activation, exhaustion, and effector fate decisions, extending beyond the canonical checkpoint receptors [70]. These networks therefore offer a crucial opportunity to elucidate mechanisms of immune resistance and rationally modulate immune cell function for cancer immunotherapy.

One primary CRISPR-mediated immune cell reprogramming strategy involves disrupting specific genes in inhibitory pathways within immune effector cells to enhance their cytotoxic capacity. For instance, using CRISPR/Cas9-mediated multiplex gene editing, universal CAR-T cells have been engineered by deleting endogenous T cell receptor components and immune checkpoint regulators to improve functional persistence in suppressive tumor environments. In glioma models, the genetically reprogrammed CAR-T cells exhibited enhanced antitumor activity and resistance to PD-1-mediated inhibition, demonstrating the potential of CRISPR-mediated perturbations of checkpoint-associated regulatory pathways to improve cancer immunotherapy. However, improved survival outcomes were observed in preclinical studies following intracerebral administration, highlighting the context-dependent nature of CRISPR-engineered immune cell efficacy [71].

In addition to TCR and PD-1 editing, CRISPR approaches have recently revealed strategies to enhance immune cell function by targeting multiple intrinsic regulators of exhaustion and survival. For example, a safer epigenetic platform that combines CRISPR-mediated editing with CRISPRoff/CRISPRon epigenetic effectors enables the simultaneous modification of numerous genes in primary T cells. Because this platform relies on mRNA, it avoids the risk of introducing DSBs DNA breaks while enabling durable regulation of the target gene expression. In preclinical adoptive transfer models, epigenetically engineered CAR-T cells generated using this platform maintained stable gene expression programs and controlled tumor growth. Together, these findings illustrate the potential of CRISPR-based multiplex epigenetic editing to enhance effector function in vivo and to improve the safety profile of immune cell engineering strategies [72].

Furthermore, the limited persistence and expansion of CAR-T cells, particularly in solid tumors, remain major obstacles in cancer immunotherapy. For instance, a particular study reported that targeted disruption of SUV39H1, a histone H3K9 methyltransferase involved in heterochromatin, reprograms human CAR-T cells to enhance functional persistence. The reprogrammed CAR-T cells exhibited sustained antitumor activity and prolonged functionality across multiple tumors rechallenges in preclinical solid tumor models. Single-cell transcriptomic analysis further revealed early reprogramming into a stem-like, self-renewing CAR-T cell population with reduced expression of genes associated with dysfunction. Together, these findings highlight the potential of targeting epigenetic regulators of immune cell fate to enhance the efficacy of adoptive cellular therapies, particularly against challenging solid tumors [73].

Collectively, these findings demonstrate that CRISPR-based immune cell reprogramming enables targeted modification in intrinsic genetic and epigenetic constraints that limit effector function and persistence. Therefore, CRISPR has evolved from a discovery tool into a robust platform for rational cellular redesign. It also offers cellular mechanistic insights that stem from functional screens that could be translated into engineered immunotherapeutic strategies. Thus, CRISPR has become a tool that maintains a continuous pipeline for not only target identification but also therapeutic implementation.

## 4. CRISPR-Based Approaches to Overcome Immune Evasion

**Figure 3:**
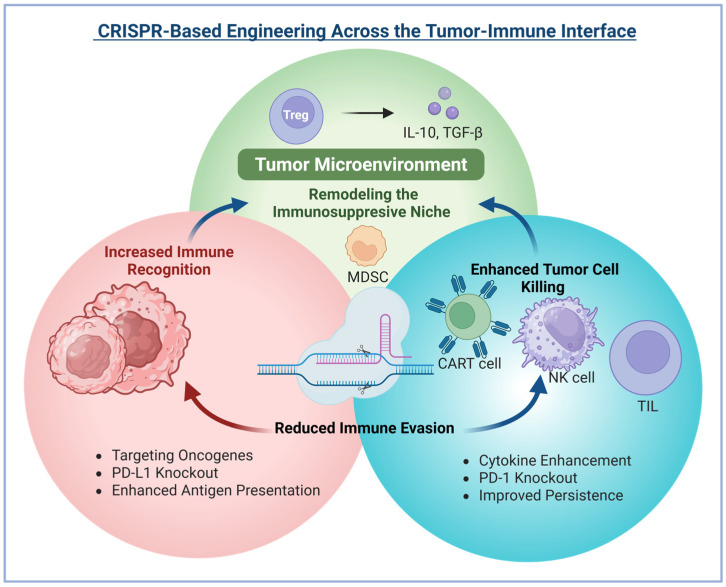
CRISPR-based engineering across the tumor-immune interface. This conceptual schematic illustrates how CRISPR interventions target three interconnected components of the tumor ecosystem: tumor cells, the tumor microenvironment (TME), and immune effector cells. Genome editing can enhance tumor immunogenicity, remodel immunosuppressive signals within the TME, and improve the cytotoxic function and persistence of engineered immune cells such as CAR T and NK cells. Created in Biorender. S. Abubakar et al. (2026) www.BioRender.com (accessed on 14 March 2026).

Cancer immunotherapy has indicated a high level of efficacy across various cancer types [74]. However, challenges such as off-target toxicity, an immunosuppressive TME, T cell exhaustion, and poor T cell quality hinder cancer immunotherapy from reaching its full potential [75]. CRISPR genome editing has enabled high-throughput identification of immune evasion mechanisms and has enabled the development of strategies to counter them (Table 1) (Figure 3). In addition to uncovering immunomodulatory genes, it has also aided in enhancing T cell activity, suppressing inhibitory receptor expression, and redirecting T cell antigen specificity by editing CARs or TCRs at the endogenous TCR-α chain (TRAC) locus [1]. This section summarizes key CRISPR strategies for overcoming immune evasion, with insights from preclinical and clinical studies.

### 4.1. Editing Immune Checkpoints

A major barrier to effective cancer immunotherapy is immune evasion, often driven by T cell exhaustion. In both human and mouse models, exhausted T cells express high levels of inhibitory receptors such as PD-1 (CD279), CTLA-4 (CD152), LAG3, TIM-3 (HAVCR2), 2B4 (CD244), CD160, and TIGIT, which impair their activation and antitumor `function [76]. CRISPR/Cas9-based genome editing offers a powerful strategy to counteract this suppression by disrupting immune checkpoint pathways and enhancing T cell responses [77]. For instance, CRISPR-mediated deletion of the PD-1 gene in primary T cells has been shown to boost their activation and cytotoxic potential. In cytotoxic T lymphocytes (CTLs), PD-1 knockdown not only improves effector function but also suppresses regulatory T cells (Tregs), further amplifying the immune response.

This strategy has shown particular promise in infection-related cancers, such as Epstein–Barr virus-associated gastric carcinoma (EBVaGC). In these tumors, LMP2A-expressing T cells exhibit elevated levels of PD-L1 (CD274) and PD-L2 (CD273). LMP2A-derived peptides can sensitize peripheral blood lymphocytes, triggering a CTL response against EBVaGC cells [78]. In addition to surface checkpoints, several intracellular regulators within effector immune cells serve as potential therapeutic targets. One example is the orphan nuclear receptor NR2F6, which functions as an intracellular immune checkpoint by repressing transcription of key cytokines such as TNFα, IL-2, and IFNγ, molecules critical for tumor rejection [32]. CRISPR-based knockout of NR2F6 has been proposed as a novel strategy to enhance T cell-mediated immunity [79].

CRISPR screening has also identified protein tyrosine phosphatase 1B (PTP1B) as a negative regulator of T cell function. PTP1B interferes with JAK/STAT signaling by dephosphorylating JAK2 and TYK2, thereby limiting T cell activation, proliferation, and cytotoxicity. Deleting PTP1B boosts STAT5 signaling, promotes antigen-driven CD8+ T cell expansion, and inhibits solid tumor growth. Notably, PTP1B inhibition has been shown to enhance the efficacy of both natural and adoptively transferred T cells including CAR-T cells and improve responses to PD-1 blockade [80]. Another relevant immune checkpoint is TIGIT, commonly expressed on antitumor T cells such as CD103^+^ tissue-resident memory (Trm) cells. TIGIT suppresses immune responses by inhibiting the costimulatory receptor CD226. It acts synergistically with PD-1, recruiting the phosphatase Shp2, which deactivates CD28 and CD226 signaling pathways, further dampening T cell function. Dual blockade of PD-L1 and TIGIT disrupts this suppressive network, restoring costimulatory signaling and enhancing antitumor T cell activity [81]. Together, these studies demonstrate that CRISPR/Cas9 can be leveraged not only to target classical surface checkpoints but also to uncover and modulate intracellular regulators. This dual targeting strategy has the potential to significantly enhance the effectiveness of cancer immunotherapy by overcoming multiple layers of immune evasion.

**Table 1:** Immunotherapy sensitizing target identified by CRISPR/Cas9 screening.

Significant Target	Target Function	Target	CRISPR/Cas9 Delivery Vector	Mechanism	Therapeutic Implication	Reference
*DDR1*	A collagen receptor involved in colorectal cancer (CRC) liver metastasis and the related stromal response	CRC cells	Lentiviral vector	Blocking *DDR1*	Promoted CD8^+^ T cell infiltration and increased the sensitivity of microsatellite stable (MSS) CRC mouse models to PD1 inhibition	[82]
*CDC7*	A serine/threonine kinase essential for DNA replication, also implicated in small-cell lung cancer (SCLC) resistance	SCLC cell line	Lentiviral vector	Knocking down *CDC7*	A lower IC50 and enhanced the effectiveness of chemotherapy	[83]
*TRIM34*	Promotes resistance to ferroptosis by inhibiting degradation of GPX4 mRNA, contributing to the progression of Hepatocellular carcinoma (HCC)	HCC cell line Huh7	Lentiviral vector	knockdown of *TRIM34*	Enhanced response of HCC cells to antiPD1 therapy	[84]
*TUBB3*	A member of the tubulin family, encodes βIIItubulin. Increased expression in tumor progression	Lung cancer cell lines (CMT167cells and C57BL/6)	Lentiviral vector	Inhibiting *TUBB3*	Cells resistant to anti-PD1 immunotherapy more vulnerable to T cell-mediated destruction by reducing the expression of PDL-1	[85]
*PTPN2*	Encodes a protein tyrosine phosphatase involved in regulating various intracellular processes, including IFNγ signaling, which it inhibits by dephosphorylating STAT1 and JAK1	Malignant melanoma cell line (A375 cells)	CuSRNP@PEI nanoparticles	Knockdown of *PTPN2*	Boosted CD8^+^ T cell infiltration and cytokine production within the tumor microenvironment, leading to a stronger immune response and increased tumor sensitivity to immunotherapy	[33]
*GDF15*	Recognized as a crucial element in immune evasion	Glioblastoma cells	angiopep2decorated, glutathione (GSH)responsive nanoparticles [ANPSS(Cas9/sgGDF15)]	Deletion of *GDF15*	Alleviated immunosuppression in the TME, enhancing the antitumor efficacy of ICB therapy	[86]
*HDAC7* and genes involved in the Sec61 pathway	A crucial transcriptional corepressor, and Sec61 is a translocon. Both demonstrated to reduce expression of BCMA, a key immunotherapy target in multiple myeloma	Multiple Myeloma cell lines	lentiviral vector	Silencing HDAC7 and Inhibiting Sec61	Increases BCMA expression, improves the antimyeloma effectiveness of a BCMA-targeted antibody–drug conjugate	[87]
*COX2*	Produces an immunosuppressive molecule prostaglandin E2 (PGE2)	Model of KRASmutant lung adenocarcinoma	Lentiviral vector	Targeting the *COX2/PGE2* signaling pathway	Enhances the response of KRAS-mutant lung tumors to antiPD1 therapy and delays tumor relapse following KRAS inhibition	[88]
*SP20H*	Upregulates the expression of B7H3 (a receptor that suppresses T cell activation) in tumor cells	Ovarian cancer cells	Lentiviral vector	Deleting the *SP20H* gene	Inhibits tumor growth, increases the ratio of CD8^+^/CD4^+^ T cells, and decreases M2 macrophage infiltration in SP20Hdeficient tumors	[89]
*Lgals2*	βgalactosidebinding lectin, modulates the immune response by regulating inflammation and immune cell interactions.	4 T1 cell lines	pCDH/Lentiviral vector	Block *Lgals2*	Effectively suppresses tumor growth and reactivates the immune system	[90]
*Lgals2*	βgalactosidebinding lectin, modulates the immune response by regulating inflammation and immune cell interactions.	HEK293 cell lines	Lentiviral vector	overexpression of *Lgals2*	Reduced the proliferation of human colon epithelial cells and diminished H2O2-induced STAT3 phosphorylation	[91]
*TPST2*	An enzyme catalyzes the sulfation of tyrosine residues in proteins. This post-transcriptional modification is essential for many cellular processes, including cell signaling and cell adhesion.	Human breast cell lines (MDAMB231 and MDA MB468) and mouse colon cancer cell line (MC38),	Lentiviral vector	Reducing *TPST2*	Increased effectiveness of anti-PD1 antibodies in syngeneic tumor models by enhancing tumor-infiltrating lymphocytes	[31]
*ATXN3*	Deubiquitinase for various PDL-1 transcription factors in tumor cells.	LLC1/B16 cells	Lentiviral vector	Specific disruption of *ATXN3*	Significant decrease in PDL-1 transcription, ultimately enhancing the immune system’s antitumor effect by working synergistically with checkpoint blockade therapy	[92]
*Ptpn 2/PDL-1*	Ptpn2 regulates intracellular process including IFNγ signaling, while PDL-1 is a ligand that interacts with program cell receptors in immune checkpoint mechanism	B1F10 cells	PX333	Disruption of *PTPN2* and PDL-1	Reinvigorates the immune system by halting the immune checkpoint effect. Additionally, the JAK/STAT pathway is reactivated following the deletion of PTPN2, which promotes the vulnerability of tumor cells to CD8^+^ T cells	[34]
*PRODH2*	An enzyme that plays an important role in proline metabolism.	HEK293FT, E0771, and MCF7 cells.	Lentiviral vector	Specific overexpression or knocking of *PRODH2*	Improved the CAR-T-based killing and the in vivo effectiveness in various cancer models	[69]
*DHX37*	Plays a key role in RNA metabolism, particularly in the regulation of gene expression	Murine breast cancer cells	Lentiviral vector	Knockout of Dhx37	Enhanced efficacy of antigen-specific CD8^+^ T cells in fighting triple-negative breast cancer in vivo	
*Cop1*	Known for its role in recruiting M2type macrophages.	4 T1 cells	Lentiviral vector	Disruption of *Cop 1*	Inhibits cancer cells’ immune escape and enhances the effectiveness of ICB	[93]
*Asf1a*	ASF1 is a histone H3H4 chaperone conserved from yeast to human cells	Lung cancer cell lines	pXPRGFPBlast vector	Inhibition of *Asf1a*	Sensitizes tumor cells to antiPD1 therapy	[94]
*KM2D*	Encodes a histone H3K4 methyltransferase, one of the genes mutated in cancer patients.	E0771, B16F10 MA1L, LLC, and MB49 cells	AAV vector	Loss of *KM2D*	Increases tumor cell sensitivity to ICB by boosting tumor immunogenicity	[95]

### 4.2. Engineering CAR-T Cells

ACT is a promising immunotherapeutic strategy that involves isolating a patient’s immune cells, genetically modifying, and expanding them ex vivo to enhance their antitumor activity and reinfusing them into the patient. ACT aims to overcome immune suppression, including poor T cell function, inadequate T cell production, and weak memory T cell development. The main types of ACT include TIL therapy, TCR-based therapy, and CAR-T cell therapy. Other evolving forms of ACT involve engineered macrophages, B cells, dendritic cells, and NK cells [96].

While it is not yet endorsed as a first-line treatment for cancers, the CAR-T cancer therapy method has revolutionized the management of certain aggressive lymphomas and multiple myelomas that have relapsed or are challenging to treat [66]. CAR-T cells combat tumors by releasing cytotoxic molecules (perforins and granzymes) and pro-inflammatory cytokines (e.g., IFN-γ, IL-2), which modify the TME and encourage apoptosis and necrosis in tumor cells [96].

CAR-T therapy has progressed through multiple generations, each built upon the previous one to improve its safety and efficacy, and several CAR-T therapies have gained FDA approval, and many are currently undergoing clinical trials [96,97,98,99,100]. Despite these advances, CAR-T therapy faces limitations. High manufacturing costs, lengthy production timelines, and difficulty in generating high-quality T cells from critically ill patients hinder broader application. Furthermore, disease relapse and variability in autologous CAR-T products contribute to inconsistent outcomes [76,101].

To address these challenges, CRISPR/Cas9 gene editing has emerged as a transformative tool to improve CAR-T safety, efficacy, and scalability. Disruption of co-inhibitory genes like PD-1 and CTLA-4 using CRISPR has been shown to enhance CAR-T cell persistence and antitumor function [102]. Researchers are also integrating CAR-T with chemotherapy, radiotherapy, and ICIs to overcome the TME’s suppressive effects, although these combinations have yet to fully resolve current limitations [103].

CRISPR screens have identified TGF-β and CD7 as critical regulators of CAR-T efficacy in solid and hematologic cancers. TGF-β, secreted by stromal and tumor-associated cells, initiates signaling via TGFBR2, which induces T cell exhaustion by upregulating immune checkpoints (e.g., PD-1, TIM-3, CTLA-4, LAG3) and promoting Treg-like phenotypes through FOXP3 expression [104]. In a study the dual knockout of PDCD1 (PD-1) and TGFBR2 further improved T cell proliferation and resistance to TME suppression in xenograft models, highlighting the value of multiplexed gene editing to enhance CAR-T efficacy [104]. In T cell malignancies, targeting CD7 poses a unique challenge since both malignant and healthy T cells express this marker, risking fratricide. To address this, researchers developed UCART7, an “off-the-shelf” CAR-T product engineered using CRISPR/Cas9 to knock out both CD7 and TRAC (T cell receptor alpha chain). UCART7 showed strong antitumor activity against T-ALL while avoiding graft-versus-host disease in preclinical models [105].

### 4.3. Engineering Tumor-Infiltrating Lymphocytes (TILs)

While CAR-T has had remarkable success in hematologic cancers, its effectiveness against solid tumors remains limited. As a result, attention has shifted toward TIL therapy, particularly for solid tumors. TILs are T cells naturally residing within tumor tissue, like CD4^+^ and CD8^+^ αβ T cells [106,107]. Unlike CAR-T therapy, which sources T cells from peripheral blood, TIL, therapy is a personalized form of immunotherapy where immune cells are isolated from a patient’s tumor, expanded, and reinfused (fig:ijms-27-02930-f004) [108]. This approach has shown meaningful benefit in some patients with advanced melanoma and non-small cell lung cancer [109,110].

Its approval for treatment-resistant melanoma in early 2024 has renewed interest in applying TIL therapy to other cancers [111,112]. However, while effective in melanoma, TIL therapy still faces challenges in other solid tumors due to a more suppressive TME and potential adverse effects like immunosuppression, infection, and organ toxicity [113]. For example, CRISPR screens have been used to identify intracellular checkpoints like CISH, which encodes cytokine-inducible SH2-containing protein, a novel intracellular immune checkpoint that function immediately downstream of the T cell receptor (TCR) to regulate functional avidity, expansion, and neoantigen reactivity, and *SOCS1*, a suppressor of cytokine signaling acts as a braking mechanism that reduces T cell activity. Preclinical studies have shown that knocking out *SOCS1* increased IFN-γ production, improved cytokine responsiveness, and enhanced the antitumor efficacy of TILs [114]. While previous studies have demonstrated the feasibility of using CRISPR/Cas9 genome editing to genetically modify TILs in order to enhance their therapeutic efficacy [113,114], CRISPR/Cas9 nucleases can introduce DSBs, which may lead to unintended genomic alterations such as translocations, inversions, and chromosomal deletions at sites of endonuclease activity, particularly in the context of multiplex genome editing. More recently, base-editing approaches have been shown to provide a safer alternative for engineering TILs. For example, adenine base editing has been used to efficiently generate single- and dual-knockouts of the inhibitory receptors TIM3 and TIGIT in TILs, resulting in enhanced cellular expansion, increased cytokine production, improved serial tumor-cell killing, and superior tumor control in vivo. Importantly, because base editing does not rely on inducing DSBs, this strategy markedly reduces the occurrence of insertion–deletion mutations and the risk of chromosomal rearrangements. These findings highlight the potential of base-editing technologies to enable safer multiplex engineering of TILs and support further exploration of additional immune checkpoint targets to improve clinical responses in adoptive cell therapies [114,115].

### 4.4. Disrupting Tumor-Intrinsic Immune Evasion Genes

The immunophenotype of the TME significantly influences the response to ICIs, underscoring the need for detailed profiling of immune signatures and the discovery of new therapeutic targets to enhance immunotherapy outcomes. CRISPR-based functional screens targeting immune-related genes have become powerful tools for identifying such targets, for instance, *SLC7A5*, a neutral amino acid transporter essential for citrulline uptake and arginine biosynthesis [116], and DYRK1B, a dual-specificity kinase that supports cell survival and proliferation [117]. Another gene, *Cop1*, an E3 ubiquitin ligase, was shown to regulate M2 tumor-associated macrophages (TAMs) and response to anti-PD-1 therapy by targeting the oncogene C/ebpδ for degradation [93]. These findings open new avenues for modulating both cancer cell survival and the immunosuppressive microenvironment.

Targeting immunosuppressive components of the TME, particularly TAMs, also holds promise. TAMs, commonly associated with poor prognosis, can be modulated using CRISPR. For example, CD47, a “don’t eat me” signal frequently overexpressed in tumors, interacts with SIRPα on macrophages to prevent phagocytosis. The CD47 was disrupted in tumor cells via CRISPR and combined with IL-12 expression, an immunostimulatory cytokine produced by macrophages and dendritic cells. Engineered tumor cells secreted IL-12, reprogramming TAMs in situ and shifting them toward a pro-inflammatory (M1) phenotype. This dual approach significantly enhanced anti-tumor immune responses and reduced tumor growth [118].

Further supporting this strategy, PGAM5, a mitochondrial phosphatase linked to M2-TAM infiltration, was recently identified as another tumor-intrinsic regulator. CRISPR/Cas9-mediated disruption of PGAM5 in tumor cells reduced M2-TAM recruitment, slowed tumor progression, and strengthened immune responses [119]. These studies highlight the therapeutic potential of targeting tumor-intrinsic genes to reshape the TME and improve immunotherapy efficacy.

### 4.5. Emerging Base Editing Platform in Natural Killer Cell and CAR-T Cell Engineering

Similar to CAR-T cells and TILs, NK cells have attracted considerable attention in cancer immunotherapy (Table 2), largely due to their inherent ability to kill transformed cells lacking major histocompatibility complexes (MHC) and antibody-bound target cells through antigen-dependent cellular cytotoxicity (ADCC). These cells have been used as a donor-derived NK cell infusion and as genetically modified cells carrying chimeric antigen receptors, known as CAR-NK cells. Compared with CAR-T cell therapies, NK cells offer safety and manufacturing advantages. including a lower risk of cytokine release syndrome, reduced neurotoxicity, and the potential for off-the-shelf manufacturing. Despite these benefits and encouraging early clinical outcomes, NK cell therapies have rarely achieved durable tumor eradication. Limited persistence, functional exhaustion, and impaired metabolic fitness remain major barriers to sustained clinical efficacy. In addition to these challenges, allogeneic products face rejection because donor and recipient Human Leucocyte Antigen (HLA) are polymorphic and usually need to be matched. Meanwhile, autologous manufacturing or donor registries are slow, labor-intensive, and expensive; so, several groups aim to create “universal” or broadly compatible donor T cells. Turning off HLA helps donor T cells avoid attack from the patient’s T cells. However, complete loss of HLA expression renders cells susceptible to NK-cell-mediated killing through missing self-recognition [120]. To mitigate this, protective inhibitory ligands such as HLA-E, which suppresses NKG2A+ NK cells, or HLA-G, proposed to inhibit KIR2DL and ILT2 expressing NK cells, can be overexpressed. While effective, each additional genetic modification increases manufacturing complexity and regulatory burden.

**Table 2:** Comparative CRISPR engineering strategies across CAR T Cells, TILs, and NK cells.

Cellular Platform	Key Advantage	Major Limitations	CRISPR-Based Solution	Reference
CAR-T cells	- High antigen specificity - Strong clinical success in hematologic malignancies	- High manufacturing costs - Lengthy production timelines - Difficulty in generating high-quality T cells from critically ill patients - Antigen escape - T cell exhaustion - Limited efficacy in solid tumors	Disruption of inhibitory genes like PD-1, CD7, TGFBR2, CTLA-4, and TRAC editing for universal CAR-T, multiplex editing to enhance persistence	[101,102,103,104,105]
TILs	- Natural tumor specificity - Polyclonal recognition of tumor antigens	- More suppressive TME - Potential adverse effects like immunosuppression, infection, and organ toxicity - Exhaustion - Variable expansion capacity - Complex manufacturing	- Knocking out or disrupting *SOCS1*, TIM3, and TIGIT enhanced cellular expansion, increased cytokine production, improved serial tumor-cell killing - Editing inhibitory receptors (PD-1, CTLA-4), enhancing metabolic fitness	[114,115]
NK cells	- Lower risk of cytokine release syndrome - Reduced neurotoxicity - The potential for off-the-shelf manufacturing - Innate cytotoxicity - Suitable for off-the-shelf therapy	- Limited persistence - Functional exhaustion - Impaired metabolic fitness - Allogeneic products face rejection - Autologous manufacturing or donor registries are slow, labor-intensive, and expensive	- Deletion of inhibitory receptors - Cytokine signaling enhancement - CAR-NK engineering - Turning off HLA	[120]

A practical alternative is to reduce HLA diversity to a minimal set of alleles that match the recipient. This preserves normal cell physiology while retaining some canonical HLA expression, which can act as a safety feature during tumorigenesis [120]. Although CRISPR-Cas9 knockout approaches can enhance NK cell activity, editing multiple genes simultaneously can lead to hazardous DNA breaks and genomic instability. Base-editing technology offers a safer and more precise alternative [121]. Base editors can induce genetic modifications without relying on DSB formation, particularly in the context of multiple gene editing. Due to their capacity for programmable introduction of a single-point mutation, base editors have been employed to facilitate gene disruption via generation of a premature termination codon (PTC) or by mutation of splice acceptor (SA) or splice donor (SD) sites with high precision and efficiency while generating minimal undesired editing outcomes compared with standard nucleases [122].

**Figure 5:**
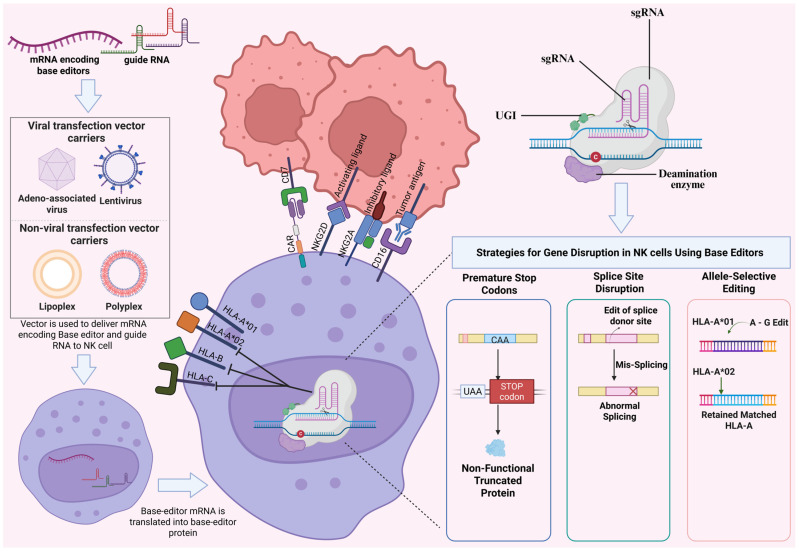
This diagram provides a comprehensive, step-by-step overview of how base editors are delivered to natural killer (NK) cells and how they are used to disrupt genes, enhancing NK cell function. It integrates delivery methods, molecular mechanisms, and functional outcomes in a single schematic. The base editor is encoded as mRNA, together with a guide RNA that specifies the genomic target. These nucleic acids are delivered using either viral vectors, such as adeno-associated virus or lentivirus, or non-viral delivery systems, including lipoplexes and polyplexes. Once inside the NK cell, the base editor mRNA is translated into a functional base editor protein. The base editor binds DNA without creating a double-strand break and performs single-nucleotide conversion within a defined editing window. Three major strategies used to disrupt genes in NK cells using base editors include:Premature stop codon introduction, whereby a single nucleotide edit converts a sense codon, such as CAA, into a stop codon UAA. This results in a truncated, non-functional protein, effectively knocking out the gene. Splice site disruption, which involves editing conserved splice donor or splice acceptor sites to causes mis-splicing. This produces abnormal mRNA transcripts and disrupts gene expression. Allele-selective editing, whereby specific HLA alleles, such as HLA-A*01, are selectively inactivated using precise base changes. A matched allele, such as HLA-A*02, is preserved. These edits improve NK cell function, persistence, and compatibility for cancer immunotherapy. Created in Biorender. S. Abubakar et al. (2026) www.BioRender.com (accessed on 13 January 2026).

This same logic enables allele-selective edits: guides can be designed to inactivate unwanted HLA-A, -B, or -C alleles while preserving one matched allele, which limits NK activation and keeps some antigen presentation intact (Figure 5) [120]. Recent work demonstrated for the first time that adenine base editing can be applied efficiently to primary human NK cells. In this study, multiplex base editing targeting up to six loci was achieved without loss of efficiency. Base editing was combined with non-viral TcBuster transposon-mediated gene integration to generate interleukin-15 armored CD19 CAR-NK cells. These engineered NK cells exhibited enhanced persistence, cytotoxicity, and resistance to immune suppression in a preclinical model of Burkitt’s lymphoma [121].

Base-editing strategies have also advanced the development of universal CAR-T cell products. In a first-in-human study, cytosine base editing was used to generate allogeneic CD7 CAR-T cells with simultaneous disruption of TRBC, CD7, and CD5. This multiplex editing strategy prevented fratricide, reduced graft versus host disease risk, and conferred resistance to alemtuzumab during lymphodepletion. Two of three treated pediatric patients achieved molecular remission within 28 days. Although multilineage cytopenia was observed, the results support further development of DSBs free multiplex editing approaches [123]. Additional research using tBE, a cytidine base-editing system, has achieved efficient knockout of TRAC, PDCD1, CD52, and B2M in primary human T cells. Notably, this approach showed reduced chromosomal abnormalities compared with Cas9 nuclease-based methods [124].

Beyond canonical base editors, modular RNA aptamer-guided Pinpoint base-editing systems have been developed to enable highly efficient and site-specific cytosine editing in human immortalized cells [125]. This aptamer-guided pinpoint base editing platform has also been used to achieve a concurrent site-specific CAR knock-in and multiplex knockouts in primary human T cells with high editing efficiency and a markedly lower translocation rate relative to CRISPR-Cas9 nuclease workflows [122].

## 5. Challenges and Considerations in CRISPR-Based Immunotherapy Development

### 5.1. Safety and Off-Target Effects

While CRISPR/Cas9 offers transformative potential in cancer immunotherapy, several challenges must be addressed to ensure its safe, scalable and clinically effective application [66]. A major concern is the risk of off-target effects, which can introduce unintended mutations and adverse consequences [126]. Furthermore, simultaneous editing at multiple genomic sites can increase the risk of chromosomal translocations or large deletions [127]. Researchers are working to enhance precision through refined delivery systems, most notably, by using CRISPR as a ribonucleoprotein (RNP) complex, which reduces integration risks and improves editing efficiency [128]. Another concern is the uncertainty surrounding long-term effects. For instance, in animal models of tyrosinemia type 1, CRISPR-mediated deletion of the *Hpd* gene corrected the metabolic disorder but unexpectedly increased hepatocellular carcinoma incidence, emphasizing the need for long-term safety assessments in cancer-prone contexts [129].

The immunogenicity of Cas proteins also represents a potential barrier to clinical translation. Many commonly used CRISPR nucleases, such as Cas9 derived from *Streptococcus pyogenes* or *Staphylococcus aureus*, originate from bacterial species to which humans may have pre-existing immune exposure [130]. This raises the possibility that edited cells expressing residual Cas proteins could be targeted by the host immune system, potentially reducing therapeutic persistence. Strategies to mitigate this issue include transient delivery of Cas9 as ribonucleoprotein complexes, the development of humanized or less immunogenic Cas variants, and the exploration of alternative editing systems such as compact CRISPR systems like Cas14 (Cas12f) [131,132]. Despite their smaller size, these nucleases initially suffered from low efficiency [133]. However, an engineered variant, eCas12f1, demonstrated potent anticancer activity by effectively targeting PLK1, a gene critical for cell cycle progression, significantly reducing colony formation in breast cancer cells [134].

### 5.2. Delivery Challenges

Delivery remains a key challenge in CRISPR therapeutics. Plasmids and viral vectors, such as lentiviruses and retroviruses, remain widely used due to their high transduction efficiency and stable gene expression. Viral vectors, such as lentiviruses and retroviruses, remain widely used due to their high transduction efficiency and stable gene expression. However, viral delivery systems carry potential drawbacks, including insertional mutagenesis, complex manufacturing requirements, and regulatory concerns [135,136]. However, the use of a rigorous guide RNA (gRNA) design process coupled with electroporation to deliver the ribonucleoprotein (RNP) complex is an emerging strategy to minimize the downstream effects of CRISPR-Cas-based gene editing. Electroporation, a method that involves applying an electrical field to cells to facilitate the delivery of genetic material, is often used in CRISPR-based systems to introduce RNP complexes directly into cells, avoiding the potential risks associated with using viral or plasmid vectors. A study by Chamberlain et al. (2022) demonstrated that electroporation could still produce clinically relevant numbers of engineered tumor-infiltrating lymphocytes (TILs) that were ready for infusion [108].

The use of lipid nanoparticles and transposon-based systems is also being increasingly explored as a safer and more flexible alternative. These methods can reduce the risk of long-term genomic integration and allow for transient exposure to CRISPR components, thereby limiting off-target activity [66,137]. Another promising delivery method involves extracellular vehicles (EVs), which are naturally compatible with the body and can cross biological barriers, such as the blood–brain barrier. EVs efficiently deliver CRISPR/Cas9 components to tumor cells and immune cells, including in hard-to-reach cancers like glioblastoma [128].

Combining CRISPR with immunotherapy and chemotherapy also offers exciting therapeutic potential. For example, a pH-sensitive nanoparticle system using PEI–PLGA was developed to co-deliver CRISPR plasmids and paclitaxel. This system not only knocked down PD-L1 expression via Cas9 targeting of Cdk5 but also enhanced immune activation and tumor suppression by inducing immunogenic cell death and shifting macrophage populations [138].

### 5.3. Ethical Considerations, Manufacturing and Regulatory Landscape

CRISPR/Cas technology is advancing at a pace that outstrips existing regulations, raising concerns about its vast yet controversial potential. In light of this, it is crucial to establish appropriate laws and develop ethical guidelines to effectively evaluate both the benefits and risks of this technology [139]. A key ethical concern surrounding CRISPR gene editing is the use of germline cells and embryo editing, which could have unforeseen consequences for future generations. These changes may lead to long-lasting effects, given the uncertainties regarding unintended outcomes beyond the intended edits [140].

Given the uncertainties surrounding CRISPR, several regulatory bodies, including the WHO, UNESCO, and the Declaration on the Human Genome and Human Rights, are working to establish guidelines. While policies vary by country, legally binding agreements such as the “Oviedo Convention” (Convention on Human Rights and Biomedicine) and regulatory agencies like the UK’s HFEA and the U.S. FDA play significant roles in shaping these regulations. The U.S. National Academy of Sciences (NAS) and National Academy of Medicine (NAM) have also emphasized the significance of engaging with the public in discussions regarding the possible advantages and negative consequences that may result from the editing of the human genome [141].

During a meeting held in the United Kingdom in 2015, it was argued that clinical research should be restricted due to fears of misuse. Despite bioethical constraints, the UK’s Authority for Human Embryology and Fertilization approved CRISPR experiments on embryos, sparking significant controversy. This approval raised concerns, particularly regarding the potential for creating genetically modified humans resistant to HIV [142].

One major risk associated with CRISPR is the possibility of unintended or off-target effects, as the single-guide RNA used by CRISPR does not require an exact match for Cas9 to induce a double-stranded break. Given the complexity of the human genome, this increases the likelihood of unintended genetic modifications, which could potentially lead to new genetic disorders, including cancer. Moreover, our incomplete understanding of human genes means that modifying one gene could unintentionally disrupt essential biological functions. Some of these unintended effects might not become apparent until future generations, adding complexity to ethical considerations. These risks have sparked global debates and led to regulatory actions, as the scientific community strives to balance CRISPR’s promise for medical advancements with ethical responsibility [143].

The production of gene-edited immune cells requires highly controlled manufacturing processes that comply with good manufacturing practice (GMP) standards. Scaling these processes while maintaining consistency, efficiency, and product safety is complex and resource intensive. Regulatory agencies also require extensive characterization of edited cells, including comprehensive assessments of editing efficiency, genomic integrity, and potential off-target effects, which can further complicate clinical development. Finally, the growing use of multiplex genome editing, in which multiple genes are simultaneously modified to enhance therapeutic function, raises important ethical and safety considerations. While multiplex editing can improve cell persistence, tumor targeting, and resistance to immunosuppressive signals, it also increases the complexity of the engineered product and the potential for unintended genomic alterations. Careful evaluation of risk–benefit profiles, transparent regulatory oversight, and continued ethical dialogue will be essential as these advanced engineering strategies move toward broader clinical use [144].

## 6. Future Directions and Innovations

The CRISPR/Cas9 system has revolutionized genetic engineering and various treatment approaches, especially in immunotherapy [145]. Although CRISPR/Cas9 holds immense therapeutic potential, concerns regarding off-target effects and immunogenicity persist, demanding safe and precise CRISPR-based therapeutics [146,147,148]. Prospective developments in the CRISPR/Cas9 system involve exploring alternative delivery strategies to mitigate immunogenic responses the engineering of Cas9 variants with improved specificity in genome editing, and optimized guide RNA configurations [149,150,151,152,153]. Additionally, the risk of unintended genomic alterations can be minimized through DNA base editing and prime editing—precision gene-editing techniques capable of making precise nucleotide changes without inducing DSBs. These techniques hold exponential promise as an instrument of therapy for controlling disease-causing mutations in the human genome in a programmable manner [146,151,154]. CRISPRi/CRISPRa can implement transcriptional regulation, engineer cellular metabolism, and elucidate genotype–phenotype mapping across smaller targeted libraries to genome-wide libraries [155]. Among the numerous advancements in CRISPR technology, CRISPRi, and CRISPRa are particularly significant for their ability to fine-tune gene expression by selectively repressing or activating immune-related genes; these tools hold significant potential for immune modulation, offering enhanced therapeutic strategies against different diseases, including cancer [155,156,157]. The CRISPR/Cas9 system has the potential to design single-treatment options to disrupt multiple mechanisms utilized by tumors to avoid targeting by the immune system. This approach of finding intervention within a single therapeutic framework promises to improve outcomes of immunotherapy, specifically where tumors resist conventional single-target therapies [52,158]. Moreover, the CRISPR/Cas9 system can help unlock immune responses that are typically suppressed in certain patients by targeting specific genetic alterations within tumor cells [159,160], which will be helpful in designing resilient cancer therapies that counteract specific immune evasion strategies [161]. The ability to modify genes in specific patient populations enables personalized therapeutic strategies, and tailored treatments according to tumor characteristics and an individual’s unique genetic makeup [1,161]. The CRISPR/Cas9 system offers unique precision in manipulating genes involved in drug metabolism, drug resistance, and individualized therapeutic effects, opening new avenues for patient-specific therapies [154,159] and advancing pharmacogenomics and personalized drug therapy [154]. The CRISPR/Cas9 system can provide a more stronger immune cells action against tumors through the modification or disruption of immune checkpoints, notably, PD-1, in T cells [162,163,164]. Similarly, the concurrent use of CRISPR/Cas9 system with cytokine therapies has been reported to provide synergistic effects, i.e., a substantial reduction in adverse side effects and enhanced anti-tumor immunity [163]. CRISPR/Cas technology has been recently exploited for the development of recombinant viral vector vaccines and live attenuated vaccines for human diseases such as certain viral infections. A novel vaccine candidate has been developed by researchers through the insertion of various viral parts within the appropriate compartments of a phage nanoparticle. This innovative nanovaccine design platform holds promise for the efficient deployment of phage-based vaccinations against a wide range of viral diseases [165,166]. Aside from its significant synergistic role in cancer treatment, immunotherapy, and personalized treatment options based on individualized genetic makeup, the CRISPR/Cas9 technology also holds promise for the treatment of addiction and familial or hereditary disorders [147,148]. Genetic influences involved in drug absorption, distribution, and metabolism have been identified as promising targets for CRISPR-based treatments in the context of addiction. The simultaneous harnessing of CRISPR/Cas9 systems with epigenetic mechanisms has been reported as a recent advancement that may allow for the creation of novel therapeutics and addiction treatments. For example, anxiety and alcoholism can be alleviated and disruptive epigenetic modifications can be reversed through the use CRISPR gene modification [147]. Anxiety disorders (ADs), major depressive disorders (MDDs), autistic spectrum disorders (ASDs), attention deficit hyperactivity disorders (ADHDs), and schizophrenia (SP) are among the most significant hereditary disorders [26,27,148,167]. Several genes have been identified as faulty and linked to these conditions, including ZNRD1, TRIM26, SYNE1, ITIH3, TCF4, DPR1, NT5C2, CACNB2, PPP1R11, CACNA1C, TENM4, CNNM2, ANK3, CSMD1, and AS3MT. These genes are mainly expressed in synaptic transmission, cellular mechanism, neuronal activity, and immune regulation, and play a pivotal role in the development of the above-mentioned disorders [168]. These genetic alterations in the affected subjects present potential targets for CRISPR/Cas9-based treatments for these disorders [26,148].

## 7. Conclusions

CRISPR/Cas9 genome editing has led to various groundbreaking biomedical research discoveries. As demonstrated by the studies highlighted in this review, its application in cancer immunotherapy has proven an effective strategy for enhancing treatment efficacy and mitigating the challenges of conventional ICB and ACT. However, before CRISPR/Cas9-modified immunotherapy can be established as a novel therapeutic strategy in oncology, extensive preclinical and clinical trials are needed for deeper insights into its safety, efficacy, and long-term effects.

## Figures and Tables

**Figure 4:**
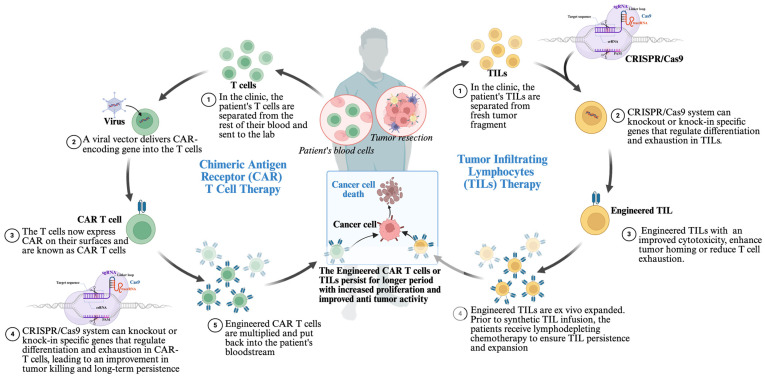
A schematic of CRISPR/Cas9 technology applications in CAR-T cells and TILs to improve long-term persistence and antitumor activity. Created in Biorender. S. Abubakar et al. (2026) www.BioRender.com (accessed on 3 March 2025).

## Data Availability

No new data were created or analyzed in this study. Data sharing is not applicable to this article.

## References

[1] Feng X., Li Z., Liu Y., Chen D., Zhou Z. (2024). CRISPR/Cas9 Technology for Advancements in Cancer Immunotherapy: From Uncovering Regulatory Mechanisms to Therapeutic Applications. Exp. Hematol. Oncol..

[2] Mellman I., Coukos G., Dranoff G. (2011). Cancer Immunotherapy Comes of Age. Nature.

[3] O’Connor M.J. (2015). Targeting the DNA Damage Response in Cancer. Mol. Cell.

[4] Sharma P., Allison J.P. (2015). The Future of Immune Checkpoint Therapy. Science.

[5] Sharma P., Allison J.P. (2020). Dissecting the Mechanisms of Immune Checkpoint Therapy. Nat. Rev. Immunol..

[6] Rafiq S., Hackett C.S., Brentjens R.J. (2020). Engineering Strategies to Overcome the Current Roadblocks in CAR T Cell Therapy. Nat. Rev. Clin. Oncol..

[7] Majzner R.G., Mackall C.L. (2018). Tumor Antigen Escape from CAR T-Cell Therapy. Cancer Discov..

[8] Kim S.K., Cho S.W. (2022). The Evasion Mechanisms of Cancer Immunity and Drug Intervention in the Tumor Microenvironment. Front. Pharmacol..

[9] Sun C., Dotti G., Savoldo B. (2016). Utilizing Cell-Based Therapeutics to Overcome Immune Evasion in Hematologic Malignancies. Blood.

[10] Ghosh D., Venkataramani P., Nandi S., Bhattacharjee S. (2019). CRISPR–Cas9 a Boon or Bane: The Bumpy Road Ahead to Cancer Therapeutics. Cancer Cell Int..

[11] Li H., Yang Y., Hong W., Huang M., Wu M., Zhao X. (2020). Applications of Genome Editing Technology in the Targeted Therapy of Human Diseases: Mechanisms, Advances and Prospects. Signal Transduct. Target. Ther..

[12] O’Driscoll M., Jeggo P.A. (2006). The Role of Double-Strand Break Repair—Insights from Human Genetics. Nat. Rev. Genet..

[13] Rouet P., Smih F., Jasin M. (1994). Expression of a Site-Specific Endonuclease Stimulates Homologous Recombination in Mammalian Cells. Proc. Natl. Acad. Sci. USA.

[14] Kaniecki K., De Tullio L., Greene E.C. (2018). A Change of View: Homologous Recombination at Single-Molecule Resolution. Nat. Rev. Genet..

[15] Verma P., Greenberg R.A. (2016). Noncanonical Views of Homology-Directed DNA Repair. Genes Dev..

[16] Chang H.H.Y., Pannunzio N.R., Adachi N., Lieber M.R. (2017). Non-Homologous DNA End Joining and Alternative Pathways to Double-Strand Break Repair. Nat. Rev. Mol. Cell Biol..

[17] Lieber M.R., Gu J., Lu H., Shimazaki N., Tsai A.G., Nasheuer H.-P. (2010). Nonhomologous DNA End Joining (NHEJ) and Chromosomal Translocations in Humans. Genome Stability and Human Diseases.

[18] Delacôte F., Lopez B.S. (2008). Importance of the Cell Cycle Phase for the Choice of the Appropriate DSB Repair Pathway, for Genome Stability Mintenance: The Trans-S Double-Strand Break Repair Model. Cell Cycle.

[19] Kim H., Kim J.-S. (2014). A Guide to Genome Engineering with Programmable Nucleases. Nat. Rev. Genet..

[20] Cui Z., Liu H., Zhang H., Huang Z., Tian R., Li L., Fan W., Chen Y., Chen L., Zhang S. (2021). The Comparison of ZFNs, TALENs, and SpCas9 by GUIDE-Seq in HPV-Targeted Gene Therapy. Mol. Ther.-Nucleic Acids.

[21] Gaj T., Gersbach C.A., Barbas C.F. (2013). ZFN, TALEN, and CRISPR/Cas-Based Methods for Genome Engineering. Trends Biotechnol..

[22] Lino C.A., Harper J.C., Carney J.P., Timlin J.A. (2018). Delivering CRISPR: A Review of the Challenges and Approaches. Drug Deliv..

[23] Cong L., Ran F.A., Cox D., Lin S., Barretto R., Habib N., Hsu P.D., Wu X., Jiang W., Marraffini L.A. (2013). Multiplex Genome Engineering Using CRISPR/Cas Systems. Science.

[24] Mali P., Yang L., Esvelt K.M., Aach J., Guell M., DiCarlo J.E., Norville J.E., Church G.M. (2013). RNA-Guided Human Genome Engineering via Cas9. Science.

[25] Cox D.B.T., Platt R.J., Zhang F. (2015). Therapeutic Genome Editing: Prospects and Challenges. Nat. Med..

[26] Allemailem K.S., Alsahli M.A., Almatroudi A., Alrumaihi F., Alkhaleefah F.K., Rahmani A.H., Khan A.A. (2022). Current Updates of CRISPR/Cas9-mediated Genome Editing and Targeting within Tumor Cells: An Innovative Strategy of Cancer Management. Cancer Commun..

[27] Allemailem K.S., Almatroodi S.A., Almatroudi A., Alrumaihi F., Al Abdulmonem W., Al-Megrin W.A.I., Aljamaan A.N., Rahmani A.H., Khan A.A. (2023). Recent Advances in Genome-Editing Technology with CRISPR/Cas9 Variants and Stimuli-Responsive Targeting Approaches within Tumor Cells: A Future Perspective of Cancer Management. Int. J. Mol. Sci..

[28] Chen B., Deng Y., Ren X., Zhao J., Jiang C. (2024). CRISPR/Cas9 Screening: Unraveling Cancer Immunotherapy’s ‘Rosetta Stone’. Trends Mol. Med..

[29] Strong A., Musunuru K. (2017). Genome Editing in Cardiovascular Diseases. Nat. Rev. Cardiol..

[30] Huber A., Djajawi T.M., Rivera I.S., Vervoort S.J., Kearney C.J. (2026). CRISPR Screens Define Unified Hallmarks of Cancer Cell-Autonomous Immune Evasion. Cell Rep..

[31] Oh Y., Kim S., Kim Y., Kim H., Jang D., Shin S., Lee S.-J., Kim J., Lee S.E., Oh J. (2024). Genome-Wide CRISPR Screening Identifies Tyrosylprotein Sulfotransferase-2 as a Target for Augmenting Anti-PD1 Efficacy. Mol. Cancer.

[32] Klepsch V., Hermann-Kleiter N., Baier G. (2016). Beyond CTLA-4 and PD-1: Orphan Nuclear Receptor NR2F6 as T Cell Signaling Switch and Emerging Target in Cancer Immunotherapy. Immunol. Lett..

[33] Yan T., Yang K., Chen C., Zhou Z., Shen P., Jia Y., Xue Y., Zhang Z., Shen X., Han X. (2021). Synergistic Photothermal Cancer Immunotherapy by Cas9 Ribonucleoprotein-Based Copper Sulfide Nanotherapeutic Platform Targeting PTPN2. Biomaterials.

[34] Yang J., Li Z., Shen M., Wang Y., Wang L., Li J., Yang W., Li J., Li H., Wang X. (2021). Programmable Unlocking Nano-Matryoshka-CRISPR Precisely Reverses Immunosuppression to Unleash Cascade Amplified Adaptive Immune Response. Adv. Sci..

[35] Li Y.-R., Halladay T., Yang L. (2024). Immune Evasion in Cell-Based Immunotherapy: Unraveling Challenges and Novel Strategies. J. Biomed. Sci..

[36] Caswell D.R., Swanton C. (2017). The Role of Tumour Heterogeneity and Clonal Cooperativity in Metastasis, Immune Evasion and Clinical Outcome. BMC Med..

[37] Rabilloud T., Potier D., Pankaew S., Nozais M., Loosveld M., Payet-Bornet D. (2021). Single-cell profiling identifies pre-existing CD19-negative subclones in a B-ALL patient with CD19-negative relapse after CAR-T therapy. Nat Commun..

[38] Mei H., Li C., Jiang H., Zhao X., Huang Z., Jin D., Guo T., Kou H., Liu L., Tang L. (2021). A Bispecific CAR-T Cell Therapy Targeting BCMA and CD38 in Relapsed or Refractory Multiple Myeloma. J. Hematol. Oncol..

[39] Zhang Z., Chen X., Tian Y., Li F., Zhao X., Liu J., Yao C., Zhang Y. (2020). Point Mutation in CD19 Facilitates Immune Escape of B Cell Lymphoma from CAR-T Cell Therapy. J. Immunother. Cancer.

[40] Orlando E.J., Han X., Tribouley C., Wood P.A., Leary R.J., Riester M., Levine J.E., Qayed M., Grupp S.A., Boyer M. (2018). Genetic Mechanisms of Target Antigen Loss in CAR19 Therapy of Acute Lymphoblastic Leukemia. Nat. Med..

[41] Ruella M., Xu J., Barrett D.M., Fraietta J.A., Reich T.J., Ambrose D.E., Klichinsky M., Shestova O., Patel P.R., Kulikovskaya I. (2018). Induction of Resistance to Chimeric Antigen Receptor T Cell Therapy by Transduction of a Single Leukemic B Cell. Nat. Med..

[42] Warda W., Da Rocha M.N., Trad R., Haderbache R., Salma Y., Bouquet L., Roussel X., Nicod C., Deschamps M., Ferrand C. (2021). Overcoming Target Epitope Masking Resistance That Can Occur on Low-Antigen-Expresser AML Blasts after IL-1RAP Chimeric Antigen Receptor T Cell Therapy Using the Inducible Caspase 9 Suicide Gene Safety Switch. Cancer Gene Ther..

[43] Hui X., Tian X., Ding S., Sun A., Zhao T., Wang H. (2025). Reprogramming the Tumor Microenvironment to Overcome Immunotherapy Resistance in Pancreatic Cancer. Front. Immunol..

[44] Xia X., Yang Z., Lu Q., Liu Z., Wang L., Du J., Li Y., Yang D.-H., Wu S. (2024). Reshaping the Tumor Immune Microenvironment to Improve CAR-T Cell-Based Cancer Immunotherapy. Mol. Cancer.

[45] Alsaafeen B.H., Ali B.R., Elkord E. (2025). Resistance Mechanisms to Immune Checkpoint Inhibitors: Updated Insights. Mol. Cancer.

[46] Li Y., Yang C., Liu Z., Du S., Can S., Zhang H., Zhang L., Huang X., Xiao Z., Li X. (2022). Integrative Analysis of CRISPR Screening Data Uncovers New Opportunities for Optimizing Cancer Immunotherapy. Mol. Cancer.

[47] Ishino Y., Shinagawa H., Makino K., Amemura M., Nakata A. (1987). Nucleotide Sequence of the Iap Gene, Responsible for Alkaline Phosphatase Isozyme Conversion in Escherichia Coli, and Identification of the Gene Product. J. Bacteriol..

[48] Jinek M., Chylinski K., Fonfara I., Hauer M., Doudna J.A., Charpentier E. (2012). A Programmable Dual-RNA–Guided DNA Endonuclease in Adaptive Bacterial Immunity. Science.

[49] Bolotin A., Quinquis B., Sorokin A., Ehrlich S.D. (2005). Clustered Regularly Interspaced Short Palindrome Repeats (CRISPRs) Have Spacers of Extrachromosomal Origin. Microbiology.

[50] Pourcel C., Salvignol G., Vergnaud G. (2005). CRISPR Elements in Yersinia Pestis Acquire New Repeats by Preferential Uptake of Bacteriophage DNA, and Provide Additional Tools for Evolutionary Studies. Microbiology.

[51] Van Der Oost J., Westra E.R., Jackson R.N., Wiedenheft B. (2014). Unravelling the Structural and Mechanistic Basis of CRISPR–Cas Systems. Nat. Rev. Microbiol..

[52] Chen C., Wang Z., Qin Y. (2023). CRISPR/Cas9 System: Recent Applications in Immuno-Oncology and Cancer Immunotherapy. Exp. Hematol. Oncol..

[53] Jiang W., Bikard D., Cox D., Zhang F., Marraffini L.A. (2013). RNA-Guided Editing of Bacterial Genomes Using CRISPR-Cas Systems. Nat. Biotechnol..

[54] Hillary V.E., Ceasar S.A. (2023). A Review on the Mechanism and Applications of CRISPR/Cas9/Cas12/Cas13/Cas14 Proteins Utilized for Genome Engineering. Mol. Biotechnol..

[55] Barrangou R., Fremaux C., Deveau H., Richards M., Boyaval P., Moineau S., Romero D.A., Horvath P. (2007). CRISPR Provides Acquired Resistance Against Viruses in Prokaryotes. Science.

[56] Deveau H., Barrangou R., Garneau J.E., Labonté J., Fremaux C., Boyaval P., Romero D.A., Horvath P., Moineau S. (2008). Phage Response to CRISPR-Encoded Resistance in *Streptococcus thermophilus*. J. Bacteriol..

[57] Sternberg S.H., Redding S., Jinek M., Greene E.C., Doudna J.A. (2014). DNA Interrogation by the CRISPR RNA-Guided Endonuclease Cas9. Nature.

[58] Sapranauskas R., Gasiunas G., Fremaux C., Barrangou R., Horvath P., Siksnys V. (2011). The Streptococcus Thermophilus CRISPR/Cas System Provides Immunity in Escherichia Coli. Nucleic Acids Res..

[59] Anders C., Niewoehner O., Duerst A., Jinek M. (2014). Structural Basis of PAM-Dependent Target DNA Recognition by the Cas9 Endonuclease. Nature.

[60] Gasiunas G., Barrangou R., Horvath P., Siksnys V. (2012). Cas9–crRNA Ribonucleoprotein Complex Mediates Specific DNA Cleavage for Adaptive Immunity in Bacteria. Proc. Natl. Acad. Sci. USA.

[61] Makarova K.S., Grishin N.V., Shabalina S.A., Wolf Y.I., Koonin E.V. (2006). A Putative RNA-Interference-Based Immune System in Prokaryotes: Computational Analysis of the Predicted Enzymatic Machinery, Functional Analogies with Eukaryotic RNAi, and Hypothetical Mechanisms of Action. Biol. Direct.

[62] Rouet P., Smih F., Jasin M. (1994). Introduction of Double-Strand Breaks into the Genome of Mouse Cells by Expression of a Rare-Cutting Endonuclease. Mol. Cell. Biol..

[63] Li G., Yang X., Luo X., Wu Z., Yang H. (2023). Modulation of Cell Cycle Increases CRISPR-Mediated Homology-Directed DNA Repair. Cell Biosci..

[64] He C., Han S., Chang Y., Wu M., Zhao Y., Chen C., Chu X. (2021). CRISPR Screen in Cancer: Status Quo and Future Perspectives. Am. J. Cancer Res..

[65] Manguso R.T., Pope H.W., Zimmer M.D., Brown F.D., Yates K.B., Miller B.C., Collins N.B., Bi K., LaFleur M.W., Juneja V.R. (2017). In Vivo CRISPR Screening Identifies Ptpn2 as a Cancer Immunotherapy Target. Nature.

[66] Yin L., He W., Wang Y., Zhang H., Huang M., Yan Y., Li S., Feng X., Saenz F., Zhang J. (2026). FACS-Based Genome-Wide CRISPR Screening Platform Identifies Modulators of CD47. Front. Immunol..

[67] Dong M.B., Wang G., Chow R.D., Ye L., Zhu L., Dai X., Park J.J., Kim H.R., Errami Y., Guzman C.D. (2019). Systematic Immunotherapy Target Discovery Using Genome-Scale In Vivo CRISPR Screens in CD8 T Cells. Cell.

[68] Lin C.-P., Levy P.L., Alflen A., Apriamashvili G., Ligtenberg M.A., Vredevoogd D.W., Bleijerveld O.B., Alkan F., Malka Y., Hoekman L. (2024). Multimodal Stimulation Screens Reveal Unique and Shared Genes Limiting T Cell Fitness. Cancer Cell.

[69] Ye L., Park J.J., Peng L., Yang Q., Chow R.D., Dong M.B., Lam S.Z., Guo J., Tang E., Zhang Y. (2022). A Genome-Scale Gain-of-Function CRISPR Screen in CD8 T Cells Identifies Proline Metabolism as a Means to Enhance CAR-T Therapy. Cell Metab..

[70] Xiang M., Li H., Zhan Y., Ma D., Gao Q., Fang Y. (2024). Functional CRISPR Screens in T Cells Reveal New Opportunities for Cancer Immunotherapies. Mol. Cancer.

[71] Choi B.D., Yu X., Castano A.P., Darr H., Henderson D.B., Bouffard A.A., Larson R.C., Scarfò I., Bailey S.R., Gerhard G.M. (2019). CRISPR-Cas9 Disruption of PD-1 Enhances Activity of Universal EGFRvIII CAR T Cells in a Preclinical Model of Human Glioblastoma. J. Immunother. Cancer.

[72] Goudy L., Ha A., Borah A.A., Umhoefer J.M., Chow L., Tran C., Winters A., Talbot A., Hernandez R., Li Z. (2025). Integrated Epigenetic and Genetic Programming of Primary Human T Cells. Nat. Biotechnol..

[73] Saitakis M. (2024). Epigenetic Reprogramming of CAR T Cells for in Vivo Functional Persistence against Solid Tumors. Genes Immun..

[74] Smyth M.J., Ngiow S.F., Ribas A., Teng M.W.L. (2016). Combination Cancer Immunotherapies Tailored to the Tumour Microenvironment. Nat. Rev. Clin. Oncol..

[75] Nishino M., Giobbie-Hurder A., Hatabu H., Ramaiya N.H., Hodi F.S. (2016). Incidence of Programmed Cell Death 1 Inhibitor–Related Pneumonitis in Patients with Advanced Cancer: A Systematic Review and Meta-Analysis. JAMA Oncol..

[76] Ou X., Ma Q., Yin W., Ma X., He Z. (2021). CRISPR/Cas9 Gene-Editing in Cancer Immunotherapy: Promoting the Present Revolution in Cancer Therapy and Exploring More. Front. Cell Dev. Biol..

[77] Allemailem K.S., Alsahli M.A., Almatroudi A., Alrumaihi F., Abdulmonem W.A., Moawad A.A., Alwanian W.M., Almansour N.M., Rahmani A.H., Khan A.A. (2023). Innovative Strategies of Reprogramming Immune System Cells by Targeting CRISPR/Cas9-Based Genome-Editing Tools: A New Era of Cancer Management. Int. J. Nanomed..

[78] Okugawa K., Itoh T., Kawashima I., Takesako K., Mazda O., Nukaya I., Yano Y., Yamamoto Y., Yamagishi H., Ueda Y. (2004). Recognition of Epstein-Barr Virus-Associated Gastric Carcinoma Cells by Cytotoxic T Lymphocytes Induced in Vitro with Autologous Lymphoblastoid Cell Line and LMP2-Derived, HLA-A24-Restricted 9-Mer Peptide. Oncol. Rep..

[79] Miftah H., Benthami H., Badou A. (2024). Insights into the Emerging Immune Checkpoint NR2F6 in Cancer Immunity. J. Leukoc. Biol..

[80] Wiede F., Lu K.-H., Du X., Zeissig M.N., Xu R., Goh P.K., Xirouchaki C.E., Hogarth S.J., Greatorex S., Sek K. (2022). PTP1B Is an Intracellular Checkpoint That Limits T-Cell and CAR T-Cell Antitumor Immunity. Cancer Discov..

[81] Banta K.L., Xu X., Chitre A.S., Au-Yeung A., Takahashi C., O’Gorman W.E., Wu T.D., Mittman S., Cubas R., Comps-Agrar L. (2022). Mechanistic Convergence of the TIGIT and PD-1 Inhibitory Pathways Necessitates Co-Blockade to Optimize Anti-Tumor CD8+ T Cell Responses. Immunity.

[82] Wu M., Ma W., Lv G., Wang X., Li C., Chen X., Peng X., Tang C., Pan Z., Liu R. (2024). DDR1 Is Identified as an Immunotherapy Target for Microsatellite Stable Colon Cancer by CRISPR Screening. npj Precis. Onc..

[83] Deng L., Yang L., Zhu S., Li M., Wang Y., Cao X., Wang Q., Guo L. (2023). Identifying CDC7 as a Synergistic Target of Chemotherapy in Resistant Small-Cell Lung Cancer via CRISPR/Cas9 Screening. Cell Death Discov..

[84] Yao F., Zhou S., Zhang R., Chen Y., Huang W., Yu K., Yang N., Qian X., Tie X., Xu J. (2024). CRISPR/Cas9 Screen Reveals That Targeting TRIM34 Enhances Ferroptosis Sensitivity and Augments Immunotherapy Efficacy in Hepatocellular Carcinoma. Cancer Lett..

[85] Zhao D., Deshpande R., Wu K., Tyagi A., Sharma S., Wu S.-Y., Xing F., O’Neill S., Ruiz J., Lyu F. (2025). Identification of TUBB3 as an Immunotherapy Target in Lung Cancer by Genome Wide in Vivo CRISPR Screening. Neoplasia.

[86] Zou C., Liu X., Wang W., He L., Yin A., Cao Z., Zhu M., Wu Y., Liu X., Ma J. (2025). Targeting GDF15 to Enhance Immunotherapy Efficacy in Glioblastoma through Tumor Microenvironment-Responsive CRISPR-Cas9 Nanoparticles. J. Nanobiotechnol..

[87] Ramkumar P., Abarientos A.B., Tian R., Seyler M., Leong J.T., Chen M., Choudhry P., Hechler T., Shah N., Wong S.W. (2020). CRISPR-Based Screens Uncover Determinants of Immunotherapy Response in Multiple Myeloma. Blood Adv..

[88] Boumelha J., de Castro A., Bah N., Cha H., de Carné Trécesson S., Rana S., Tomaschko M., Anastasiou P., Mugarza E., Moore C. (2024). CRISPR-Cas9 Screening Identifies KRAS-Induced COX2 as a Driver of Immunotherapy Resistance in Lung Cancer. Cancer Res..

[89] Zhao S., Wang Y., Yang N., Mu M., Wu Z., Li H., Tang X., Zhong K., Zhang Z., Huang C. (2022). Genome-Scale CRISPR–Cas9 Screen Reveals Novel Regulators of B7-H3 in Tumor Cells. J. Immunother. Cancer.

[90] Ji P., Gong Y., Jin M., Wu H., Guo L.-W., Pei Y.-C., Chai W.-J., Jiang Y.-Z., Liu Y., Ma X.-Y. (2022). In Vivo Multidimensional CRISPR Screens Identify *Lgals2* as an Immunotherapy Target in Triple-Negative Breast Cancer. Sci. Adv..

[91] Li H., Zhao L., Lau Y.S., Zhang C., Han R. (2021). Genome-Wide CRISPR Screen Identifies LGALS2 as an Oxidative Stress-Responsive Gene with an Inhibitory Function on Colon Tumor Growth. Oncogene.

[92] Wang S., Iyer R., Han X., Wei J., Li N., Cheng Y., Zhou Y., Gao Q., Zhang L., Yan M. (2023). CRISPR Screening Identifies the Deubiquitylase ATXN3 as a PD-L1–Positive Regulator for Tumor Immune Evasion. J. Clin. Investig..

[93] Wang X., Tokheim C., Gu S.S., Wang B., Tang Q., Li Y., Traugh N., Zeng Z., Zhang Y., Li Z. (2021). In Vivo CRISPR Screens Identify the E3 Ligase Cop1 as a Modulator of Macrophage Infiltration and Cancer Immunotherapy Target. Cell.

[94] Li F., Huang Q., Luster T.A., Hu H., Zhang H., Ng W.-L., Khodadadi-Jamayran A., Wang W., Chen T., Deng J. (2020). In Vivo Epigenetic CRISPR Screen Identifies *Asf1a* as an Immunotherapeutic Target in *Kras*-Mutant Lung Adenocarcinoma. Cancer Discov..

[95] Wang G., Chow R.D., Zhu L., Bai Z., Ye L., Zhang F., Renauer P.A., Dong M.B., Dai X., Zhang X. (2020). CRISPR-GEMM Pooled Mutagenic Screening Identifies KMT2D as a Major Modulator of Immune Checkpoint Blockade. Cancer Discov..

[96] Hu Q., Xuan J., Wang L., Shen K., Gao Z., Zhou Y., Wei C., Gu J. (2025). Application of Adoptive Cell Therapy in Malignant Melanoma. J. Transl. Med..

[97] Goyco Vera D., Waghela H., Nuh M., Pan J., Lulla P. (2024). Approved CAR-T Therapies Have Reproducible Efficacy and Safety in Clinical Practice. Hum. Vaccines Immunother..

[98] Commissioner O. FDA Approves CAR-T Cell Therapy to Treat Adults with Certain Types of Large B-Cell Lymphoma. https://lymphoma.org/news/fda-approves-first-cart-therapy-for-lymphoma/.

[99] Leick M.B., Maus M.V., Frigault M.J. (2021). Clinical Perspective: Treatment of Aggressive B Cell Lymphomas with FDA-Approved CAR-T Cell Therapies. Mol. Ther..

[100] Sengsayadeth S., Savani B.N., Oluwole O., Dholaria B. (2022). Overview of Approved CAR-T Therapies, Ongoing Clinical Trials, and Its Impact on Clinical Practice. eJHaem.

[101] Tao R., Han X., Bai X., Yu J., Ma Y., Chen W., Zhang D., Li Z. (2024). Revolutionizing Cancer Treatment: Enhancing CAR-T Cell Therapy with CRISPR/Cas9 Gene Editing Technology. Front. Immunol..

[102] Dötsch S., Svec M., Schober K., Hammel M., Wanisch A., Gökmen F., Jarosch S., Warmuth L., Barton J., Cicin-Sain L. (2023). Long-Term Persistence and Functionality of Adoptively Transferred Antigen-Specific T Cells with Genetically Ablated PD-1 Expression. Proc. Natl. Acad. Sci. USA.

[103] Agarwal S., Aznar M.A., Rech A.J., Good C.R., Kuramitsu S., Da T., Gohil M., Chen L., Hong S.-J.A., Ravikumar P. (2023). Deletion of the Inhibitory Co-Receptor CTLA-4 Enhances and Invigorates Chimeric Antigen Receptor T Cells. Immunity.

[104] Tang N., Cheng C., Zhang X., Qiao M., Li N., Mu W., Wei X.-F., Han W., Wang H. TGF-β Inhibition via CRISPR Promotes the Long-Term Efficacy of CAR T Cells against Solid Tumors. https://insight.jci.org/articles/view/133977/pdf.

[105] Cooper M.L., Choi J., Staser K., Ritchey J.K., Devenport J.M., Eckardt K., Rettig M.P., Wang B., Eissenberg L.G., Ghobadi A. (2018). An “off-the-Shelf” Fratricide-Resistant CAR-T for the Treatment of T Cell Hematologic Malignancies. Leukemia.

[106] Presti D., Dall’Olio F.G., Besse B., Ribeiro J.M., Di Meglio A., Soldato D. (2022). Tumor Infiltrating Lymphocytes (TILs) as a Predictive Biomarker of Response to Checkpoint Blockers in Solid Tumors: A Systematic Review. Crit. Rev. Oncol. Hematol..

[107] Zhao Y., Deng J., Rao S., Guo S., Shen J., Du F., Wu X., Chen Y., Li M., Chen M. (2022). Tumor Infiltrating Lymphocyte (TIL) Therapy for Solid Tumor Treatment: Progressions and Challenges. Cancers.

[108] Chamberlain C.A., Bennett E.P., Kverneland A.H., Svane I.M., Donia M., Met Ö. (2022). Highly Efficient PD-1-Targeted CRISPR-Cas9 for Tumor-Infiltrating Lymphocyte-Based Adoptive T Cell Therapy. Mol. Ther.-Oncolytics.

[109] Turcotte S., Donia M., Gastman B., Besser M., Brown R., Coukos G., Creelan B., Mullinax J., Sondak V.K., Yang J.C. (2025). Art of TIL Immunotherapy: SITC’s Perspective on Demystifying a Complex Treatment. J. Immunother. Cancer.

[110] Schoenfeld A.J., Lee S.M., Doger de Spéville B., Gettinger S.N., Häfliger S., Sukari A., Papa S., Rodríguez-Moreno J.F., Graf Finckenstein F., Fiaz R. (2024). Lifileucel, an Autologous Tumor-Infiltrating Lymphocyte Monotherapy, in Patients with Advanced Non–Small Cell Lung Cancer Resistant to Immune Checkpoint Inhibitors. Cancer Discov..

[111] Chesney J., Lewis K.D., Kluger H., Hamid O., Whitman E., Thomas S., Wermke M., Cusnir M., Domingo-Musibay E., Phan G.Q. (2022). Efficacy and Safety of Lifileucel, a One-Time Autologous Tumor-Infiltrating Lymphocyte (TIL) Cell Therapy, in Patients with Advanced Melanoma after Progression on Immune Checkpoint Inhibitors and Targeted Therapies: Pooled Analysis of Consecutive Cohorts of the C-144-01 Study. J. Immunother. Cancer.

[112] Kwong M.L.M., Yang J.C. (2024). Lifileucel: FDA-Approved T-Cell Therapy for Melanoma. Oncologist.

[113] Commissioner O. FDA Approves First Cellular Therapy to Treat Patients with Unresectable or Metastatic Melanoma. https://www.fda.gov/news-events/press-announcements/fda-approves-first-cellular-therapy-treat-patients-unresectable-or-metastatic-melanoma.

[114] Schlabach M.R., Lin S., Collester Z.R., Wrocklage C., Shenker S., Calnan C., Xu T., Gannon H.S., Williams L.J., Thompson F. (2023). Rational Design of a SOCS1-Edited Tumor-Infiltrating Lymphocyte Therapy Using CRISPR/Cas9 Screens. J. Clin. Investig..

[115] Hafezi M., Genolet R., Hadadi L., Chap B.S., Bobisse S., Giordano Attianese G.M.P., El Jorfi H., Cropp D., Pericou L., Arnaud M. (2025). Highly Efficient Gene Knockout in Tumor-Infiltrating Lymphocytes by Adenine Base Editing. Mol. Ther. Oncol..

[116] Dunlap K.N., Bender A., Bowles A., Bott A.J., Tay J., Grossmann A.H., Rutter J., Ducker G.S. (2025). SLC7A5 Is Required for Cancer Cell Growth under Arginine-Limited Conditions. Cell Rep..

[117] Rashidi A., Füchtbauer E.-M., Vahabzadeh Z., Soleimani F., Rahimi K., Nikkhoo B., Fakhari S., Erfan M.B.K., Azarnezhad A., Pooladi A. (2025). CRISPR/Cas9-Mediated Knockout of DYRK1B in Triple-Negative Breast Cancer Cells: Implications for Cell Proliferation, Apoptosis, and Therapeutic Sensitivity. Biochem. Eng. J..

[118] Lin M., Yang Z., Yang Y., Peng Y., Li J., Du Y., Sun Q., Gao D., Yuan Q., Zhou Y. (2022). CRISPR-Based in Situ Engineering Tumor Cells to Reprogram Macrophages for Effective Cancer Immunotherapy. Nano Today.

[119] Wei X., Wang H., Liu H., Wang J., Zhou P., Li X., He Y., Li Y., Han D., Mei T. (2025). Disruption of Tumor-Intrinsic PGAM5 Increases Anti-PD-1 Efficacy through the CCL2 Signaling Pathway. J. Immunother. Cancer.

[120] Winterhalter P.M., Warmuth L., Hilgendorf P., Schütz J.M., Dötsch S., Tonn T., Cicin-Sain L., Busch D.H., Schober K. (2024). HLA Reduction of Human T Cells Facilitates Generation of Immunologically Multicompatible Cellular Products. Blood Adv..

[121] Wang M., Krueger J.B., Gilkey A.K., Stelljes E.M., Kluesner M.G., Pomeroy E.J., Skeate J.G., Slipek N.J., Lahr W.S., Claudio Vázquez P.N. (2025). Precision Enhancement of CAR-NK Cells through Non-Viral Engineering and Highly Multiplexed Base Editing. J. Immunother. Cancer.

[122] Porreca I., Blassberg R., Harbottle J., Joubert B., Mielczarek O., Stombaugh J., Hemphill K., Sumner J., Pazeraitis D., Touza J.L. (2024). An Aptamer-Mediated Base Editing Platform for Simultaneous Knockin and Multiple Gene Knockout for Allogeneic CAR-T Cells Generation. Mol. Ther..

[123] Chiesa R., Georgiadis C., Syed F., Zhan H., Etuk A., Gkazi S.A., Preece R., Ottaviano G., Braybrook T., Chu J. (2023). Base-Edited CAR7 T Cells for Relapsed T-Cell Acute Lymphoblastic Leukemia. N. Engl. J. Med..

[124] Li P., Su Q., Xu Y., He J., Wang L., Wang Y., Li H., Lan K., Zheng H., Zhu D. (2023). Multiplex Genome Editing of Human T Cells with Innovative Transformer Base Editor (tBE) for Construction of Next Generation CAR-T Therapies. Blood.

[125] Collantes J.C., Tan V.M., Xu H., Ruiz-Urigüen M., Alasadi A., Guo J., Tao H., Su C., Tyc K.M., Selmi T. (2021). Development and Characterization of a Modular CRISPR and RNA Aptamer Mediated Base Editing System. CRISPR J..

[126] Sherazi S.A.M., Rafique F., Haris M., Arshad A., Qaiser H., Uzair M., Arshad M. (2023). Applications of CRISPR Cas-9 in Ovarian Cancer Research. Protein Pept. Lett..

[127] Aussel C., Cathomen T., Fuster-García C. (2025). The Hidden Risks of CRISPR/Cas: Structural Variations and Genome Integrity. Nat. Commun..

[128] Liu X., Liu X., Luo X., Zhu M., Liu N., Li J., Zhang Q., Zou C., Wu Y., Cao Z. (2025). Synergistic Strategies for Glioblastoma Treatment: CRISPR-Based Multigene Editing Combined with Immune Checkpoint Blockade. J. Nanobiotechnol..

[129] Chen T., Barzi M., Furey N., Kim H.R., Pankowicz F.P., Legras X., Elsea S.H., Hurley A.E., Yang D., Wheeler D.A. (2025). CRISPR/Cas9 Gene Therapy Increases the Risk of Tumorigenesis in the Mouse Model of Hereditary Tyrosinemia Type I. JHEP Rep..

[130] Wagner D.L., Amini L., Wendering D.J., Burkhardt L.-M., Akyüz L., Reinke P., Volk H.-D., Schmueck-Henneresse M. (2019). High Prevalence of Streptococcus Pyogenes Cas9-Reactive T Cells within the Adult Human Population. Nat. Med..

[131] Takeda S.N., Nakagawa R., Okazaki S., Hirano H., Kobayashi K., Kusakizako T., Nishizawa T., Yamashita K., Nishimasu H., Nureki O. (2021). Structure of the Miniature Type V-F CRISPR-Cas Effector Enzyme. Mol. Cell.

[132] Stigzelius V., Cavallo A.L., Chandode R.K., Nitsch R. (2025). Peeling Back the Layers of Immunogenicity in Cas9-Based Genomic Medicine. Mol. Ther..

[133] Harrington L.B., Burstein D., Chen J.S., Paez-Espino D., Ma E., Witte I.P., Cofsky J.C., Kyrpides N.C., Banfield J.F., Doudna J.A. (2018). Programmed DNA Destruction by Miniature CRISPR-Cas14 Enzymes. Science.

[134] Park S.-J., Ju S., Jung W.J., Jeong T.Y., Yoon D.E., Lee J.H., Yang J., Lee H., Choi J., Kim H.S. (2025). Robust Genome Editing Activity and the Applications of Enhanced Miniature CRISPR-Cas12f1. Nat. Commun..

[135] Yi Q., Ouyang X., Zhu G., Zhong J. (2024). Letter: The Risk-Benefit Balance of CRISPR-Cas Screening Systems in Gene Editing and Targeted Cancer Therapy. J. Transl. Med..

[136] Mao K., Tan H., Cong X., Liu J., Xin Y., Wang J., Guan M., Li J., Zhu G., Meng X. (2025). Optimized Lipid Nanoparticles Enable Effective CRISPR/Cas9-Mediated Gene Editing in Dendritic Cells for Enhanced Immunotherapy. Acta Pharm. Sin. B.

[137] Alsaiari S.K., Eshaghi B., Du B., Kanelli M., Li G., Wu X., Zhang L., Chaddah M., Lau A., Yang X. (2024). CRISPR–Cas9 Delivery Strategies for the Modulation of Immune and Non-Immune Cells. Nat. Rev. Mater..

[138] Tu K., Deng H., Kong L., Wang Y., Yang T., Hu Q., Hu M., Yang C., Zhang Z. (2020). Reshaping Tumor Immune Microenvironment through Acidity-Responsive Nanoparticles Featured with CRISPR/Cas9-Mediated Programmed Death-Ligand 1 Attenuation and Chemotherapeutics-Induced Immunogenic Cell Death. ACS Appl. Mater. Interfaces.

[139] Piergentili R., Del Rio A., Signore F., Umani Ronchi F., Marinelli E., Zaami S. (2021). CRISPR-Cas and Its Wide-Ranging Applications: From Human Genome Editing to Environmental Implications, Technical Limitations, Hazards and Bioethical Issues. Cells.

[140] Stefanoudakis D. (2025). Integrating CRISPR Technology with Key Genetic Markers in Pancreatic Cancer: A New Frontier in Targeted Therapies. SynBio.

[141] Cribbs A.P., Perera S.M.W. (2017). Science and Bioethics of CRISPR-Cas9 Gene Editing: An Analysis Towards Separating Facts and Fiction. Yale J. Biol. Med..

[142] Gonzalez-Avila L.U., Vega-López J.M., Pelcastre-Rodríguez L.I., Cabrero-Martínez O.A., Hernández-Cortez C., Castro-Escarpulli G. (2021). The Challenge of CRISPR-Cas Toward Bioethics. Front. Microbiol..

[143] Morris D., Morris D. (2024). CRISPR Technology for Adenocarcinoma: Treatment of Pancreatic Cancer. WSEAS Trans. Biol. Biomed..

[144] Wienert B., Cromer M.K. (2022). CRISPR Nuclease Off-Target Activity and Mitigation Strategies. Front. Genome Ed..

[145] Vimal S., Madar I.H., Thirumani L., Thangavelu L., Sivalingam A.M. (2024). CRISPR/Cas9: Role of Genome Editing in Cancer Immunotherapy. Oral Oncol. Rep..

[146] Kantor A., McClements M., MacLaren R. (2020). CRISPR-Cas9 DNA Base-Editing and Prime-Editing. Int. J. Mol. Sci..

[147] Akyürek E., Uysal B., Gülden G., Taştan C. (2021). The Effect Of Molecular Genetic Mechanisms On Drug Addiction And Related New Generation CRISPR Gene Engineering Applications. Gene Ed..

[148] Singh M., Agarwal V., Jindal D., Pancham P., Agarwal S., Mani S., Tiwari R.K., Das K., Alghamdi B.S., Abujamel T.S. (2023). Recent Updates on Corticosteroid-Induced Neuropsychiatric Disorders and Theranostic Advancements through Gene Editing Tools. Diagnostics.

[149] Naeem M., Majeed S., Hoque M.Z., Ahmad I. (2020). Latest Developed Strategies to Minimize the Off-Target Effects in CRISPR-Cas-Mediated Genome Editing. Cells.

[150] Lopes R., Prasad M.K. (2024). Beyond the Promise: Evaluating and Mitigating off-Target Effects in CRISPR Gene Editing for Safer Therapeutics. Front. Bioeng. Biotechnol..

[151] Uddin F., Rudin C.M., Sen T. (2020). CRISPR Gene Therapy: Applications, Limitations, and Implications for the Future. Front. Oncol..

[152] Chen P., Zhou J., Liu H., Zhou E., He B., Wu Y., Wang H., Sun Z., Paek C., Lei J. (2024). Engineering of Cas12a Nuclease Variants with Enhanced Genome-Editing Specificity. PLoS Biol..

[153] Kovalev M.A., Davletshin A.I., Karpov D.S. (2024). Engineering Cas9: Next Generation of Genomic Editors. Appl. Microbiol. Biotechnol..

[154] He J., Biswas R., Bugde P., Li J., Liu D.-X., Li Y. (2022). Application of CRISPR-Cas9 System to Study Biological Barriers to Drug Delivery. Pharmaceutics.

[155] Call S.N., Andrews L.B. (2022). CRISPR-Based Approaches for Gene Regulation in Non-Model Bacteria. Front. Genome Ed..

[156] Bendixen L., Jensen T.I., Bak R.O. (2023). CRISPR-Cas-Mediated Transcriptional Modulation: The Therapeutic Promises of CRISPRa and CRISPRi. Mol. Ther..

[157] Laufer B.I., Singh S.M. (2015). Strategies for Precision Modulation of Gene Expression by Epigenome Editing: An Overview. Epigenet. Chromatin.

[158] Djajawi T.M., Wichmann J., Vervoort S.J., Kearney C.J. (2024). Tumor Immune Evasion: Insights from CRISPR Screens and Future Directions. FEBS J..

[159] Mathur S., Sutton J. (2017). Personalized Medicine Could Transform Healthcare. Biomed. Rep..

[160] Molla G., Bitew M. (2024). Revolutionizing Personalized Medicine: Synergy with Multi-Omics Data Generation, Main Hurdles, and Future Perspectives. Biomedicines.

[161] Youssef E., Fletcher B., Palmer D. (2025). Enhancing Precision in Cancer Treatment: The Role of Gene Therapy and Immune Modulation in Oncology. Front. Med..

[162] Park H., Kang Y.K., Shim G. (2024). CRISPR/Cas9-Mediated Customizing Strategies for Adoptive T-Cell Therapy. Pharmaceutics.

[163] Caforio M., Iacovelli S., Quintarelli C., Locatelli F., Folgiero V. (2024). GMP-Manufactured CRISPR/Cas9 Technology as an Advantageous Tool to Support Cancer Immunotherapy. J. Exp. Clin. Cancer Res..

[164] Nguyen T.T.T., Greene L.A., Mnatsakanyan H., Badr C.E. (2024). Revolutionizing Brain Tumor Care: Emerging Technologies and Strategies. Biomedicines.

[165] Zahedipour F., Zahedipour F., Zamani P., Jaafari M.R., Sahebkar A. (2024). Harnessing CRISPR Technology for Viral Therapeutics and Vaccines: From Preclinical Studies to Clinical Applications. Virus Res..

[166] Zhu J., Ananthaswamy N., Jain S., Batra H., Tang W.-C., Lewry D.A., Richards M.L., David S.A., Kilgore P.B., Sha J. (2021). A Universal Bacteriophage T4 Nanoparticle Platform to Design Multiplex SARS-CoV-2 Vaccine Candidates by CRISPR Engineering. Sci. Adv..

[167] Bray N.J., O’Donovan M.C. (2018). The Genetics of Neuropsychiatric Disorders. Brain Neurosci. Adv..

[168] Barešić A., Nash A.J., Dahoun T., Howes O., Lenhard B. (2020). Understanding the Genetics of Neuropsychiatric Disorders: The Potential Role of Genomic Regulatory Blocks. Mol. Psychiatry.

